# The Role of Surgery in Global Health: Analysis of United States Inpatient Procedure Frequency by Condition Using the Global Burden of Disease 2010 Framework

**DOI:** 10.1371/journal.pone.0089693

**Published:** 2014-02-26

**Authors:** John Rose, David C. Chang, Thomas G. Weiser, Nicholas J. Kassebaum, Stephen W. Bickler

**Affiliations:** 1 Department of Surgery, University of California San Diego, La Jolla, California, United States of America; 2 Center for Surgery and Public Health, Brigham and Women’s Hospital, Boston, Massachusetts, United States of America; 3 Department of Surgery, Stanford University, Palo Alto, California, United States of America; 4 Institute for Health Metrics and Evaluation, University of Washington, Seattle, Washington, United States of America; 5 Department of Anesthesiology/Pain Medicine, University of Washington, Seattle, Washington, United States of America; 6 Division of Pediatric Surgery, Rady Children’s Hospital, San Diego, California, United States of America; University of Louisville, United States of America

## Abstract

**Background:**

The role of surgical care in promoting global health is the subject of much debate. The Global Burden of Disease 2010 study (GBD 2010) offers a new opportunity to consider where surgery fits amongst global health priorities. The GBD 2010 reinforces the DALY as the preferred methodology for determining the relative contribution of disease categories to overall global burden of disease without reference to the likelihood of each category requiring surgery. As such, we hypothesize that the GBD framework underestimates the role of surgery in addressing the global burden of disease.

**Methods and Findings:**

We compiled International Classification of Diseases, Version 9, codes from the United States Nationwide Inpatient Sample from 2010. Using the primary diagnosis code for each hospital admission, we aggregated admissions into GBD 2010 disease sub-categories. We queried each hospitalization for a major operation to determine the frequency of admitted patients whose care required surgery. Major operation was defined according to the Agency for Healthcare Research and Quality (AHRQ). In 2010, 10 million major inpatient operations were performed in the United States, associated with 28.6% of all admissions. Major operations were performed in every GBD disease subcategory (range 0.2%–84.0%). The highest frequencies of operation were in the subcategories of Musculoskeletal (84.0%), Neoplasm (61.4%), and Transport Injuries (43.2%). There was no disease subcategory that always required an operation; nor was there any disease subcategory that never required an operation.

**Conclusions:**

Surgical care cuts across the entire spectrum of GBD disease categories, challenging dichotomous traditional classifications of ‘surgical’ versus ‘nonsurgical’ diseases. Current methods of measuring global burden of disease do not reflect the fundamental role operative intervention plays in the delivery of healthcare services. Novel methodologies should be aimed at understanding the integration of surgical services into health systems to address the global burden of disease.

## Introduction

During the past ten years there has been increasing interest in defining the role of surgical care amongst other global health priorities. [1] Not long ago, surgery was referenced as the “neglected stepchild” of global health and considered amongst other “neglected diseases”. [2], [3] However, it is now generally accepted that a significant burden of disease requiring surgical intervention exists globally with 234 million operations performed each year. [4]–[8] Approximately two billion people in low- and middle-income countries (LMICs) lack access to emergency and essential surgical care and there is growing evidence of the role of surgery in achieving the Millennium Development Goals. [9]–[12] As countries struggle to create comprehensive health care packages more research is needed to determine the role of surgery in addressing national disease burdens and in the broader global health enterprise. [13]–[14].

Historically, surgical initiatives have occupied multiple places within the practice of global health. Longstanding successes include vertical initiatives to treat isolated conditions such as cleft palate and cataracts, amongst others. [15]–[19] More recently, the World Health Organization (WHO) Emergency and Essential Surgical Care program released Surgical Care at the District Hospital to incorporate essential surgical care into existing ‘horizontal’ frontline infrastructure. [20]–[22] After the release of the GBD 1996 study, Debas and colleagues made an initial estimate of the global burden of ‘surgical disease’. [23], [24] In absence of data, they identified disease categories frequently requiring surgery and estimated that 11% of global DALYs could be treated with surgery. As the era of health systems strengthening evolves, diagonal planning predominates with the aim of coordinating care across previous silos to maximize value, exemplified by the WHO Safe Surgery Saves Lives campaign. [25]–[29].

In December 2012, *The Lancet* published the Global Burden of Diseases, Injuries and Risk Factors 2010 Study (GBD 2010), which contains the most detailed and rigorous estimates of death and disability throughout the world. [30] The scope has grown from previous versions to include 291 diseases and injuries, 1160 sequelae of these conditions, and 67 risk factors in 187 countries, 20 age groups, for both sexes and with trends from 1990 to 2010. [31] Beyond mere reporting of incidence and prevalence, the GBD 2010 reinforces the use of Disability Adjusted Life Years (DALYs), Years Lived with Disability (YLDs), and Years of Life Lost due to premature mortality (YLLs) as critical tools in comparing relative disease burdens. Results are widely available with the goal that policy-makers will direct limited resources preferentially towards those therapies that address disease categories with the highest scores. [32]–[34] In this context it is imperative to determine the appropriate application of surgical care within existing health care systems. [35].

The WHO identifies nation states as the principle unit of disease burden analysis and health system strategizing. [36]–[38] Country-specific reporting from GBD 2010 enables national ministries of health to view reports of the shifting disease epidemiology within their countries and plan accordingly. [39]–[41] For example, in Mozambique the largest increases in DALYs from 1990–2010 in both sexes of all ages were HIV/AIDS (27,704%) and Road Injury (153%). In the United States during the same period, the DALYs associated with HIV/AIDS and Road Injury declined 61% and 16%, respectively, and the greatest increase was in Alzheimer’s Disease (159%). Estimations of disease burden are key for national ministries of health to develop targeted care packages and subsequently align health system performance measures to health care reform. [42]–[44].

The GBD 2010 offers a new opportunity to consider where surgical care fits amongst global health priorities and its potential impact on global public health. Using the GBD 2010 framework as a model, we assessed the role of surgery in addressing a single country’s burden of disease. In this report, we apply operative data from hospitalizations in the United States to the GBD 2010 framework to calculate the proportion of admitted patients whose care required a surgical procedure. Due to diverse epidemiologic patterns throughout the world, the U.S. example cannot be understood as representative of all countries, but was chosen given publicly available nationwide databases to test the hypothesis that surgery plays a prominent role in addressing multiple disease sub-categories. Finally, we discuss the inherent challenges of estimating the global burden of ‘surgical disease’ and offer potential steps forward to quantify the role of surgical care in the diagnosis and treatment of disease.

## Methods

This study queried the United States Nationwide Inpatient Sample (NIS) for International Classification of Diseases, Version 9 (ICD-9) codes during 2010. [45] The NIS is the largest all-payer inpatient care database in the United States, containing data from approximately 1,000 hospitals. The database is a 20% representative sample of inpatient records from 45 states. The NIS is our only source of data and comprises a publicly available de-identified patient database. As such, our study did not involve human subjects or animals. In the NIS, each hospitalization is recorded with demographic information for the patient being admitted and ICD-9 codes to describe the diagnoses and procedures that are associated with the hospital stay. Up to 30 ICD-9 codes may be associated with each admission, 15 diagnostic codes and 15 procedure codes. These were chosen because ICD-9 is the primary recording system in the United States.

All hospitalizations in 2010 were queried and grouped by GBD 2010 subcategories using the primary diagnostic ICD-9 code associated with the corresponding hospital admission. [Table 1] In this way, each primary diagnostic code corresponds to one inpatient admission. Our primary unit of analysis is thus ‘inpatient admissions’ and not persons. This accounts for each patient’s ability to be admitted on multiple occasions during the study period. The remaining non-primary ICD-9 diagnostic codes associated with each admission were not included in the analysis because we assumed that the primary diagnostic code was the principle criteria for admission to the hospital. The GBD 2010 ICD-9 codes were extracted from Table 4 of the Supplement material supplied by Lozano et al, 2012. [Appendix S1] [46].

**Table 1:** Hospital admissions and frequency of operations by GBD disease subcategories.

GBD Disease Category	Total Admissions	Associated Operations	Frequency of Operation (%)
**I. Communicable, Maternal, Neonatal and** **Nutritional Disorders**			
HIV/AIDS and tuberculosis	77582	6913	8.9
Diarrhea, LRI, meningitis, and other commoninfectious diseases	1368682	23160	1.7
Neglected tropical diseases and malaria	2888	175	6.1
Maternal disorders	3741380	1252751	33.5
Neonatal disorders	126213	19423	15.4
Nutritional deficiencies	140642	3341	2.4
Other communicable, maternal, neonataland nutritional disorders	95186	18974	19.9
*Subtotal*	*5552573*	*1324737*	*23.9*
**II. Non-Communicable Diseases**			
Neoplasms	1103714	677427	61.4
Cardiovascular and circulatory diseases	2971335	974938	32.8
Chronic respiratory diseases	1308943	56622	4.3
Cirrhosis of the liver	106616	7037	6.6
Digestive diseases (except cirrhosis)	2463959	892867	36.2
Neurological disorders	477253	48900	10.2
Mental and behavioral disorders	1738365	3511	0.2
Diabetes, urogenital, blood, and endocrine diseases	2327079	774692	33.3
Musculoskeletal disorders	1798545	1510402	84.0
Other non-communicable diseases	788880	174415	22.1
*Subtotal*	*15084689*	*5120811*	*33.9*
**III. Injuries**			
Transport injuries	197923	85465	43.2
Unintentional injuries other than transport injuries	4237055	1514734	35.7
Self-harm and interpersonal violence	290761	41209	14.2
Forces of nature, war, and legal intervention	21236	2336	11.0
*Subtotal*	*4746975*	*1643744*	*34.6*
**Uncaptured Codes**	9464108	1884817	19.9
**Totals**	34848345	9974109	28.6

All major inpatient operations in 2010 were queried and grouped into GBD 2010 disease subcategories according to the associated primary code for the same hospital encounter. In this study, major operation was defined as surgical procedures performed in the operating room on inpatients. This definition and corresponding ICD-9 procedure codes are standardized and publicly available through the Agency for Healthcare Research and Quality (AHRQ). [Appendix S2] [47] It is worth noting that the NIS includes up to 15 procedure codes for each hospital admission and our analysis did not include minor surgical procedures that take place outside the operating room, diagnostic procedures, or outpatient procedures. These measures of surgical volume were excluded because standardized criteria do not exist to define them and the procedures are not consistently linked to a diagnosis. This omission of surgical volume was considered acceptable for the current study because our hypothesis was to evaluate the relationship between the national burden of disease and surgical care and not the volume of surgery itself.

Principle outcomes included descriptive reporting of the volume of disease burden in the United States as captured by GBD 2010 primary diagnostic ICD-9 codes for inpatient admissions. The national volume of inpatient operations was also tabulated according to AHRQ ICD-9 codes for major operations. [Appendix S2] We then calculated the percentage of inpatients undergoing a major operation for each GBD 2010 disease sub-category by dividing the number of inpatient admissions associated with a major operation for a given primary diagnosis code by the total number of admissions having the same code. These numbers were then weighted to account for the database sample size of 20% with a United States population of 308,745,538 people in 2010, obtained from the US Census Bureau. [48]–[50] It is worth noting that because the NIS database does not specify which of the 15 possible diagnosis codes corresponds to which of the 15 possible procedure codes, it is impossible to ascertain the exact cause of surgery. As such, for each admission, we assume that the primary diagnosis code is related to the major operation performed. We also did not measure the prevalence of multiple operations for a single admission because it was not necessary to test our hypothesis. In addition, there is no method to control for the factors that might lead to reoperation or distinguish reoperation from two operations performed during the same visit to the operating room, making any conclusion from such data very suspect at best.

## Results

Our analysis shows a large volume of major operations performed during hospitalizations in the North American sub-region. [Table 1] In 2010, 10 million major procedures were performed in the operating room in association with 34.8 million hospital admissions. This amounts to more than one inpatient procedure in every four hospital admissions or one in every 31 people living in the United States. (U.S.) The volume of inpatient procedures across the three broad GBD disease categories was: 1.3 million for Communicable/Maternal/Neonatal/Nutritional Diseases, 5.1 million for Non-communicable Diseases, and 1.6 million for Injury. At the sub-category level, the volume varied from 175 operations for the Neglected Tropical Diseases and Malaria to a high of 1.5 million for Unintentional Injuries. The sub-categories with the largest volume of surgery were Unintentional Injuries (1.5 million), Musculoskeletal Disorders (1.5 million), and Maternal Disorders (1.3 million).

**Figure 1:**
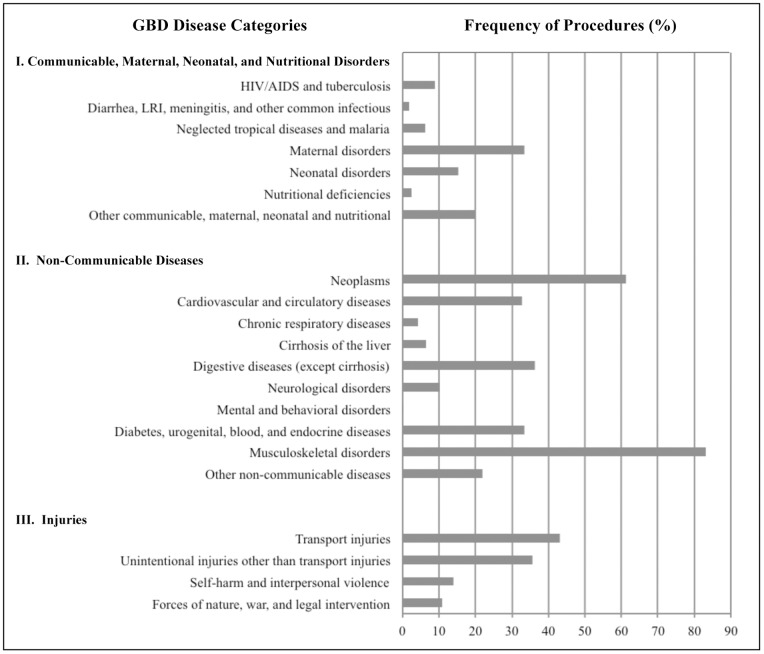
Percentage of inpatients undergoing a surgical procedure by disease subcategories.

The most important finding in our analysis is that surgical care cuts across every disease sub-category of the GBD 2010 study. [Figure 1] The overall frequency of major operation among inpatients with GBD primary diagnosis codes was 28.6%. The frequency of operation across the three broad categories of disease were; 23.9% for Communicable/Maternal/Neonatal/Nutritional Diseases, 33.9% for Non-communicable Diseases, and 34.6% for Injury. At the sub-category level, this frequency varied from 0.2% for Mental and Behavioral Disorders to a high of 84.0% for Musculoskeletal Disorders. The highest frequencies were in the Musculoskeletal (84.0%), Neoplasm (61.4%), Transport Injuries (43.2%), and Digestive Diseases (36.2%) sub-categories. There was no disease sub-category that required an operation 100% of the time. Nor was there any disease sub-category that never required an operation.

There were an estimated 9.5 million admissions with ICD-9 diagnostic codes that were either not included in the GBD 2010 Study or not assigned disability weight. This group represents 2,848 unique ICD-9 primary diagnosis codes collectively associated with 1.9 million procedures in the operating room or 19.9% of all inpatient operations during the year 2010. The two most common uncaptured diagnoses were single live birth without cesarean section and single live birth with cesarean section. While childbirth is a precarious process in many settings, the resultant newborn’s ‘admission’ to the hospital is not weighted with intrinsic disability in the GBD framework. Others included wastebasket terms that could not reliably be coded with disability scores: septicemia nos, chest pain nec, congestive heart failure nos, rehabilitation procedure nec, acute kidney failure nos, dehydration, syncope and collapse, and acute on chronic systolic heart failure. [Table 2].

**Table 2:** Top ten most frequent diagnoses not captured by GBD 2010 primary diagnosis codes.

Uncaptured Primary Diagnosis Codes (ICD-9)	Percentage of Uncaptured Admissions
Single live birth, no cesarean section (V30.00)	23.6
Single live birth with cesarean section (V30.01)	11.2
Septicemia nos (038.9)	4.9
Chest pain nec (786.59)	3.9
Congestive heart failure nos (428.0)	3.1
Rehabilitation proc nec (V57.89)	3.1
Acute kidney failure nos (584.9)	3.0
Dehydration (276.51)	2.0
Syncope and collapse (780.2)	1.9
Acute on chronic systolic heart failure (428.23)	1.9
*All Uncaptured*	*100%*

## Discussion

These data confirm that hospitalized patients in the United States frequently require major operations. Without referencing the likelihood that each disease category might require surgery, the GBD 2010 falls short of describing the applicability of surgery in promoting health at national, regional, and global levels. This study demonstrates the inherent challenges of defining the role of surgery in addressing the global burden of disease and reinforces the integration of surgical care delivery into health systems planning.

**Figure 2:**
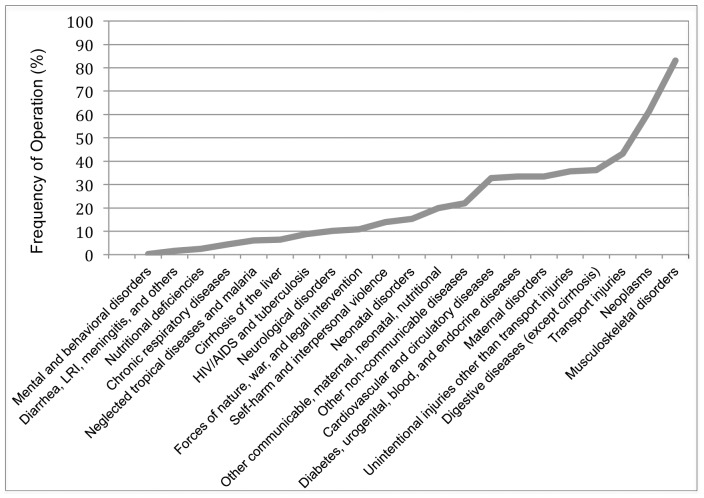
Continuum of operation frequency by disease subcategory.

Specifically, these data call into question the popular dichotomy between ‘surgical’ and ‘nonsurgical’ conditions. [51] For example, disease sub-categories of Mental and Behavioral Disorders, Digestive Diseases, and Musculoskeletal Disorder display increasing frequency of operation (0.2%, 36.2%, and 84.0%, respectively), yet there is no consensus on where to draw a line between ‘nonsurgical’ and ‘surgical’ disease categories. [Figure 2] Also consider two sub-categories with similar frequencies of operation; Maternal Disorders (33.5%) and Diabetes, Urogenital, and Endocrine Disorders (33.3%). While maternal disorders (i.e. obstructed labor, hemorrhage) are frequently discussed as ‘surgical’ in nature, the latter category is not routinely grouped as the same. [52]–[53] To the extent that the GBD 2010 framework is structured conceptually around discrete disease entities (i.e. diabetes, diarrhea, and maternal disorders) without reference to the likelihood of each category requiring operative intervention, it falls short of elucidating the role of surgery in global health.

Instead of attempting to classify diseases as surgical or non-surgical it might be more appropriate to view surgical care as an integral component of a system of care. It is important to point out that there was no disease category that never required an operation. Also, consider the sub-category of ‘Neoplasm’, where 55.8% of patients admitted for a neoplasm diagnosis underwent a surgical procedure. Certainly, there would be disagreement that all patients with a neoplasm be classified as surgical patients. Yet surgical care plays an important role in the diagnosis (biopsy), treatment (resection) and supportive care (chronic intravenous access) of patients with tumors. This again disrupts the false dichotomy between ‘surgical’ and ‘nonsurgical’ disease categories and more accurately reflects the multiple roles that surgical care plays in a broad spectrum of clinical problems. In order to meet a population’s disease burden, surgical care cannot be reduced to a vertical intervention. Instead, global health practitioners should work closely with national ministries of health to participate in broad discussions of resource management, health system financing, and efficacy of available care, as was recently described by surgical teams in Rwanda. [54]–[56].

One strength of this study is that we used a standardized list of surgical procedures compared to previous estimates of surgical volume. Debas and colleagues defined an operation as “anything that requires suture, incision, excision, manipulation, or other invasive procedures that usually, but not always, require local, regional, or general anesthesia”. [24] Weiser et al and Semel et al measured the United States nationwide operative volume by using a list of 2520 procedures generated by a consensus panel of expert surgeons. [57]–[58] An advantage of our approach is that there is a published list of what counts as a surgical procedure, removing one of the subjective components of previous calculations. In order to facilitate future calculations of surgical volume at country, regional, and global scales, it is necessary to standardize objective definitions of surgery that lend themselves to quantification with available coding systems.

Furthermore, the AHRQ procedures list allowed us to uncover a fundamental problem in disease burden measurements that rely on disability weighting. As mentioned in our methods, the NIS database associates ICD-9 diagnostic codes with ICD-9 surgical procedure codes during a given hospitalization. Using the AHRQ list of ICD-9 procedure codes, our study illustrates that the GBD 2010 Study incompletely captures patients who need surgery. In fact, 19.9% of procedures in the operating room were performed on inpatients with a primary diagnosis code not assigned disability weights in the GBD framework. Consider the most common uncaptured codes of single live birth, with or without cesarean section. However, the GBD framework assigns no disability weight to the process of giving birth (i.e. gaining life) and as such neglects the crucial role of surgery in preventing complications of labor to the fetus. Other uncaptured surgeries are those associated with primary diagnostic codes that are wastebasket terms such as ‘septicemia not otherwise specified’, which cannot be assigned disability weights despite being coded in hospital settings. For these reasons, any methodology reliant solely upon disability weighting neglects a significant proportion of associated surgical procedures.

There are several limitations of this study; the most obvious being that our approach does not include a true measure of surgical volume in the United States. The volume of surgery in the U.S. has been quantified previously by counting inpatient operative cases. Weiser et al reported 13.7 million procedures in the National Hospital Discharge Survey during 2006. [57] Semel et al used the same procedure list to query the NIS database during the same year and reported 14.3 million inpatient surgical procedures. [58] Our reported case volume of 10 million represents a conservative underestimate as it does not account for multiple procedures during the same hospitalization, bedside surgical procedures (i.e. incision and drainage, excisional biopsies, tracheostomy), diagnostic procedures (interventional radiology or cardiology), or outpatient surgical procedures (i.e. laparoscopic cholecystectomy, tonsillectomy). Of note, outpatient surgical procedures may account for as much as 30–50% of overall surgical volume, as certain procedures are increasingly performed in ambulatory surgical centers in the U.S. [59]–[60] Beyond counting cases, the current study also neglects the increasing role of non-operative management of diseases where surgical teams are involved (i.e. blunt trauma and emergency consultation). [61]–[63] For these reasons, our results grossly underestimate surgical volume or burden in the United States and do not provide an immediate answer to the question of where all surgical care fits amongst other global health priorities.

There are also inherent challenges in estimating the impact of surgical care that our study does not address. The AHRQ list includes ICD-9 procedure codes for over 2500 major operations. [Appendix S2] [47] DALYs for surgical procedures appear in the literature for a limited number of operations, but to calculate DALYs averted it is necessary to know the disability weight associated with a particular condition and the effectiveness of an operation. [64]–[69] The effectiveness of an operation varies by the type of operation, resources available to conduct the operation, operative skills or volume of the surgeon, and patient factors such as nutritional status and other co-morbidities. [70]–[72] The large number of surgical procedures combined with the variability in operative outcomes makes for a difficult, if not impossible, calculation. These and other criticisms of applying DALY calculations to surgical care were recently discussed by Gosselin et al. [73] It is also difficult to interpret inpatient operative rates in the context of the GBD prevalence data. GBD involves “counts of cases”, not “counts of people”. As such, the GBD population may have multiple diagnoses per individual. Expressing prevalence data in this fashion may be useful when attempting to measure the impact of individual diseases, but complicates the evaluation of impact that interventions have at a population level.

Another weakness lies in using the United States as an example of association between primary diagnosis and associated procedure frequency, both of which change over time. [74] The effect of ongoing epidemiological transitions was discussed earlier, and the United States disease burden cannot be understood as representative of the burden of inpatient disease worldwide. For example, the burden of Musculoskeletal Diseases – where we report the highest frequency of operations and second-highest total operative volume – rose substantially in the U.S. between 1990–2010. [75] More specifically, in 1990 Low Back Pain was associated with 2.5 million DALYs. [Table 3] By 2010 that number rose to 3.2 million, a 25% increase. The GBD group at IHME currently ranks Low Back Pain as the third highest contributor to overall DALYs in the U.S. During the same time period, randomized controlled trials showing improved outcomes with surgical decompression of spinal stenosis led to a sharp rise in operations to treat Low Back Pain. [76]–[77] Subsequently, surgical practice patterns in the U.S. favored spinal procedures with higher reoperation rates. [78]–[79] Given these unique trends in disease epidemiology and treatment availability, disease prevalence and treatment patterns in the United States cannot be immediately generalized to other countries. Additionally, Weiser et al also published large disparities in surgical volume across regions and sub-regions of the world. [8] Using health funding per capita, they reported that the wealthiest 30.2% of the world’s population receives 73.5% of global surgical volume while the poorest 34.8% receives 3.5%. While this may result from relative depravity in LMICs given known surgeon shortages, it could also be explained by relative overutilization in high-income countries or variability in disease epidemiology, such as diverticular disease. [80]–[83] Our study makes no attempt to resolve these possible explanations, except that by restricting our analysis to the inpatient setting we hope to exclude procedures that are not imminently critical for preservation of life. Nonetheless, because the United States is not inherently representative of appropriate operative volume for all countries, our results should not be generalized to a global scale. Lastly, the U.S. NIS database used here records ICD-9 codes whereas many countries have transitioned to ICD-10 coding. The GBD 2010 lists ICD-10 aggregations but cross-country comparisons were beyond the scope of our analysis. Instead of serving as a one-size-fits-all model, our study is intended to promote structured investigation of the role of surgery in each country given the known advantages of shared learning in international comparisons. [84]–[85].

**Table 3:** Summary of trends in GBD DALYs in the United States for the Musculoskeletal Disease Subcategory with corresponding rank amongst all other diseases for 1990 and 2010.

Diseases	1990*	Rank	2010*	Rank	Median %Change
Low back pain	2.5	6	3.2	3	+25
Other musculoskeletal	2.1	8	3.0	6	+43
Neck pain	1.7	10	2.1	11	+29
Osteoarthritis	0.6	31	1.0	25	+56
Rheumatoid arthritis	0.3	44	0.4	43	+28

*DALYs reported in millions.

Given the challenges outlined above, we are not optimistic that existing data appropriately estimate the role of surgical care in addressing the global burden of disease. In our dataset, surgical procedures were performed in every disease sub-category of the GBD 2010 study. Considering the current obstacles to estimating a global burden of diseases treatable with surgery, we recommend avoiding traditional dichotomies between surgical and nonsurgical disease categories and expanding the purview of the GBD analysis and other prioritization frameworks to include standardized and quantifiable definitions of surgical procedures. New strategies should also be developed for estimating the impact of integrating surgical care within the growing literature on health systems. [86]–[88] A detailed analysis of the types of diagnoses associated with operations but not assigned disability weighting in the GBD 2010 Study could also guide this effort. Using a database where unique identifiers are used for both inpatients and outpatients could also improve our ability to estimate the surgical care needed for a population. More research is also needed to determine the volume of non-operative surgical management and consultation.

## Supporting Information

Appendix S1 **ICD-9 codes from the GBD 2010 study.**
(PDF)

Appendix S2 **AHRQ ICD-9 Procedure Codes.**
(PDF)
