# Supplementary material for: The Role of Surgery in Global Health: Analysis of United States Inpatient Procedure Frequency by Condition Using the Global Burden of Disease 2010 Framework
Source: PLoS One. 2014 Feb 26;9(2):e89693. doi: 10.1371/journal.pone.0089693 (PMC3935922; doi:10.1371/journal.pone.0089693)
Supplement: Appendix S1 — ICD-9 codes from the GBD 2010 study. (PDF) [file pone.0089693.s001.pdf]

Appendix S1. ICD-9 codes from the GBD 2010 study.

GBD Disease Sub-Category	ICD-9 Codes
HIV/AIDS and tuberculosis	010-018.99 137.0-137.49 320.4-320.49 730.4-730.69 042-044.99 112-118.99 130-130.99 136.3-136.89 176.0-176.99 279-279.99
Diarrhea, LRI, meningitis, and other common infectious diseases	001-001.99 003-004.99 006-009.99 487-487.99 481-481.99 482.2-482.29 480.1-480.19 466-466.99 480.0-480.09 480.2-480.99 482.0-482.19 482.3-482.99 483-486.99 513-513.99 770.0-770.09 460-465.99 476-476.99 320.1-320.09 320.0-320.09 036-036.99 320.5-320.59 047-049.99 320.2-320.39 320.7-320.99 321-322.99
Neglected tropical diseases and malaria	084-084.99 086.0-086.09 086.2-086.29 085-085.99 086.3-086.59 120-120.99 123.1-123.19 122-122.99 125.0-125.39 076-076.99 077.0-077.09 061-061.99 060-060.99 071-071.99 126-126.99 121-121.99 127.0-127.09 127.3-127.39 126-126.99 123.0-123.09 123.2-124.99 127.1-127.29 127.4-127.99 128-129.99
Maternal disorders	630-677.99
Neonatal disorders	761.0-761.19 765-765.99 769-769.99 770.2-770.99 772.1-772.19 774.2-774.29 776.6-776.69 777.5-777.69 772.2-772.29 760-760.99 761.7-761.99 762-762.99 763-763.99 766-766.99 767-767.99 768.4-768.99 770.1-770.19 775.0-775.09 775.1-775.19 775.6-775.69 779.0-779.29 771.4-771.89 761.2-761.69 764-764.99 770.0-770.09 771-771.09 772.0-772.09 772.3-772.99 774.0-774.09 774.1-774.19 774.3-774.79 775.2-775.59 775.7-775.99 776.0-776.59 776.7-776.99 777.0-777.49 777.8-777.99 778-778.99 779.4-779.89
Nutritional deficiencies	260-263.99 243-243.99 244.2-244.29 264-264.99 280-280.99 285.9-285.99 265-269.99 281-281.99
Other communicable, maternal, neonatal and nutritional disorders	090-097.99 098-098.99 099.1-099.19 099.4-099.59 099.0-099.09 099.2-099.39 099.8-099.99 614-616.49 070-070.21 070.24-070.31 070.34-070.43 070.45-070.48 070.5-070.53 070.55-070.58 070.6-070.99 030-030.99 020-027.99 031-031.99 034-035.99 039-039.99 040.1-041.99 046-046.99 051-051.99 054-054.99 057-057.99 073-075.99 077.1-077.99 078.5-078.89 100-104.99 136.0-136.29 136.9-136.99 323.0-323.99 730.9-730.99 771.1-771.29 050-050.99 072-072.99 056-056.99 771.0-771.09 045-045.99 138-138.99 323.2-323.29 730.7-730.79
Neoplasms	150-150.99 230.1-230.29 151-151.99 235.2-235.39 155-155.99 161-161.99 235.6-235.69 162-162.99 231.1-231.29 231.8-231.89 235.7-235.79 174-175.79 233-233.99 238.3-238.39 239.3-239.39 180-180.99 233.1-233.19 182-182.99 185-185.99 233.4-233.49 236.5-236.59 153-154.99 230.3-230.69 140-145.99 235.0-235.19 146-148.99 156-157.99 172-173.99 232-232.99 238.2-238.29 183.0-183.09 236.2-236.29 186-186.99 236.4-236.49 189.0-189.29 188-188.99 233.7-233.79 236.70-236.79 239.4-239.49 191-192.99 237.0-237.19 237.5-237.99 239.6-239.69 193-193.99 200-208.99 152-152.99 158-158.99 160-160.99 163-164.99 170-171.99 181-181.99 183.2-184.89 187.0-187.89 189.3-189.89 190-190.99 194-194.99 196-198.99 230.7-230.79 233.5-233.59 234.0-234.09 234.8-234.89 235.8-235.89 236.1-235.19 237.2-237.49 238.0-238.19 210-217.99 219-231.99 236-236.99 238.4-239.29
Cardiovascular and circulatory diseases	390-391.99 392.0-392.99 393-398.99 410-414.99 430-432.99 437.2-427.29 433-435.99 437-437.19 437.5-437.89 402-402.99 422.00-422.99 425.00-425.99 427.30-427.39 441.00-441.99 443.00-443.99 421.00-421.99 392.00-392.99 416.00-417.99 420-420.99 423-423.99 423.1-424.99 427-427.29 427.6-427.99 442-442.99 446-451.99 453-455.99 456.3-459.99 435-435.99 437.6-437.79
Chronic respiratory diseases	490-492.89 494-494.99 496-496.99 500-506.99 493-493.99 135-135.99 515-516.99 470-475.99 477-478.99 495-495.99 506-506.99 508-508.99 517-519.99
Cirrhosis of the liver	070.22-070.23 070.32-070.33 070.44-070.49 070.54-070.59 456.0-456.29 571-571.99 572.3-572.89 573.9-573.99
Digestive diseases (except cirrhosis)	531-533.99 535-535.69 540-542.99 560-560.99 550-551.03 552-552.03 553-553.03 553.9-553.99 555-556.99 558-558.99 557-557.99 574-576.99 577-577.9 520-520.99 524-527.99 528-528.99 529-529.99 530-530.99 534-534.99 536-537.99 543-543.99 551.1-551.99 552.1-552.99 553.1-553.89 562-566.99 569-570.99 573-573.29 579-579.99
Neurological disorders	290-290.99 330-331.99 332-332.99 345-345.99 340-340.99 346-346.99 307.81 324-326.99 333-337.99 341-341.99 357-357.99 348-348.99 349.0-349.89 350-359.99
Mental and behavioral disorders	295-295.99 291-291.99 303-303.99 305.0-305.09 304.0-304.09 305.5-305.59 304.2-304.29 305.6-305.69 304.4-304.49 305.7-305.79 304.3-304.39 305.2-305.29 292-292.99 304.1-304.19 304.5-304.99 305.1-305.19 305.3-305.49 305.8-305.99 296.2-296.39 300.4-300.49 296.0-296.19 296.4-296.99 300.0-300.09 300.2-300.39 308-308.99 307.1-307.19 307.50-307.59 314.0-314.09 317-319.99 293-294.99 297-298.99 299.01-299.99 300.1-300.19 300.5-300.99 301.0-301.19 301.3-301.99 302-302.99 306-307.99 307.2-307.49 307.6-307.99 309-313.99 314.1-316.99 E860.0-E860.1 E850.0-E850.2 E850.9-E850.9 E851.0 E852.0-E852.5 E852.8-E852.9 E853.0-E853.2 E853.8-E853.9 E854.0-E853.3
Diabetes, urogenital, blood, and endocrine diseases	250.0-250.39 250.5-250.99 580-580.99 250.4-250.49 403-404.99 581-583.99 589-589.99 590-590.99 595-595.99 596.9-596.99 597-597.99 599.0-599.9 592-592.99 594-594.99 788.0-788.09 600-600.99 606-606.99 591-591.99 593-593.99 596-596.99 598-598.99 599.1-599.89 601-605.99 607-607.99 608-608.99 218-218.99 256.4-256.49 628-628.99 617-617.99 618-618.99 625.4-625.49 282.0-282.19 282.7-285.89 282.2-282.39 282.5-282.69 282.4-282.49 240-242.99 244-244.19 244.3-244.99 245-246.99 251-256.39 256.5-259.99 270-273.99 275-275.99 277-278.99 286-286.59 286.7-289.99 610-610.99 611-611.99 616.5-616.99 619-624.99 625.0-625.39 625.5-625.99 626-627.99 629-629.99 282.0-282.19 282.7-285.89 282.2-282.39 282.5-282.69 282.4-282.49

Musculoskeletal disorders	714-714.99 715-715.99 722-722.99 724-724.99 274-274.99 710-711.99 712-713.99 716-716.99 717-719.99 720-721.99 723-723.99 725-729.99 730.3-730.39 730.9-730.99 731-733.99 734-736.99 737-737.99 738-739.99
Other non-communicable diseases	740-741.99 742.2-742.99 745-747.99 749-749.99 758.0-758.99 758.1-758.99 742.0-742.19 743-744.99 748-748.99 750-753.99 756-759.89 690-692.99 696-696.99 681-681.99 682-682.99 680-680.99 683-683.99 684-684.99 686-686.99 707.1-707.19 707.8-707.89 707.9-707.99 133.0-133.09 110-110.99 111-111.99 078-078.99 706.0-706.99 706.1-706.19 704.0-704.09 698-698.99 708-708.99 707-707.99 685-685.99 693-693.99 694-694.99 695-695.99 697-697.99 700-700.99 701-701.99 702-706.99 709-709.99 365-365.99 366-366.99 362.5-362.59 367-368.99 378-378.99 385-385.99 387-387.99 389-389.99 361-362.59 362.6-364.99 369-369.99 360-360.99 370-377.99 379-380.99 384-384.99 386-386.99 388-388.99 521-522.99 523-523.99 798.0-798.09
Transport injuries	E800.3 E801.3 E802.3 E803.3 E804.3-E804.4 E805.3-E806.3 E810.0-E810.6 E811.0-E811.1 E811.2-E811.3 E811.4-E811.7 E812.0-E812.1 E812.2-E812.3 E812.4-E812.7 E813.0-E813.7 E814.0-E814.7 E815.0-E815.7 E816.0-E816.1 E816.2-E816.7 E817.0-E817.1 E817.2-E817.7 E818.0-E818.1 E818.2-E818.7 E819.0-E819.7 E820.0-E820.6 E821.0-E821.6 E822.0-E822.7 E823.0-E823.7 E824.0-E824.7 E825.0-E825.7 E826.0-E826.4 E827.0-E827.0 E827.2-E827.4 E828.0 E828.4 E829.0 E829.4
Unintentional injuries other than transport injuries	E830-E830.9 E832-E832.9 E850.3-E850.8 E855-E858.9 E860.2-E861.9 E862-E886.9 E888-E888.9 E890-E899.9 E900.1 E901.1 E902-E903.9 E904.1-E904.9 E905-E906.9 E910-E928.2 E928.8 E930-E949.9
Self-harm and interpersonal violence	E904-E904.9 E950-E950.9 E951-E955.5 E955.9 E956-E969.9
Forces of nature, war, and legal intervention	E900.0 E900.9 E901.0 E901.8 E901.9 E907-E909.9 E990-E999.9 E970-E978.9
