# Supplementary material for: The Role of Surgery in Global Health: Analysis of United States Inpatient Procedure Frequency by Condition Using the Global Burden of Disease 2010 Framework
Source: PLoS One. 2014 Feb 26;9(2):e89693. doi: 10.1371/journal.pone.0089693 (PMC3935922; doi:10.1371/journal.pone.0089693)
Supplement: Appendix S2 — AHRQ ICD-9 Procedure Codes. (PDF) [file pone.0089693.s002.pdf]

Appendix S2. AHRQ ICD-9 Procedure Codes.

0044 PROC-VESSEL BIFURCATION OCT06-<br>0050 IMPL CRT PACEMAKER SYS<br>0051 IMPL CRT DEFIBRILLAT SYS<br>0052 IMP/REP LEAD LF VEN SYS<br>0053 IMP/REP CRT PACEMAKR GEN<br>0054 IMP/REP CRT DEFIB GENAT<br>0056 INS/REP IMPL SENSOR LEAD OCT06-<br>0057 IMP/REP SUBCUE CARD DEV OCT06-<br>0061 PERC ANGIO PRECEREB VES (OCT 04)<br>0062 PERC ANGIO INTRACRAN VES (OCT 04)<br>0066 PTCA OR CORONARY ATHER OCT05-<br>0070 REV HIP REPL-ACETAB/FEM OCT05-<br>0071 REV HIP REPL-ACETAB COMP OCT05-<br>0072 REV HIP REPL-FEM COMP OCT05-<br>0073 REV HIP REPL-LINER/HEAD OCT05-<br>0074 HIP REPL SURF-METAL/POLY OCT05-<br>0075 HIP REP SURF-METAL/METAL OCT05-<br>0076 HIP REP SURF-CERMC/CERMC OCT05-<br>0077 HIP REPL SURF-CERMC/POLY OCT06-<br>0080 REV KNEE REPLACEMT-TOTAL OCT05-<br>0081 REV KNEE REPL-TIBIA COMP OCT05-<br>0082 REV KNEE REPL-FEMUR COMP OCT05-<br>0083 REV KNEE REPLACE-PATELLA OCT05-<br>0084 REV KNEE REPL-TIBIA LIN OCT05-<br>0085 RESRF HIPTOTAL-ACET/FEM OCT06-<br>0086 RESRF HIPPART-FEM HEAD OCT06-<br>0087 RESRF HIPPART-ACETABLUM OCT06-<br>0112 OPEN CEREB MENINGES BX<br>0114 OPEN BRAIN BIOPSY<br>0115 SKULL BIOPSY<br>0118 OTHER BRAIN DX PROCEDURE<br>0119 OTHER SKULL DX PROCEDURE<br>0121 CRANIAL SINUS I & D<br>0122 REMOV INTRACRAN STIMULAT<br>0123 REOPEN CRANIOTOMY SITE<br>0124 OTHER CRANIOTOMY<br>0125 OTHER CRANIECTOMY<br>0126 INS CATH-CRANIAL CAVITY OCT05-<br>0127 REM CATH-CRANIAL CAVITY OCT05-<br>0128 INTRACEREB CTH-BURR HOLE OCT06-<br>0131 INCISE CEREBRAL MENINGES<br>0132 LOBOTOMY & TRACTOTOMY<br>0139 OTHER BRAIN INCISION<br>0141 THALAMUS OPERATIONS<br>0142 GLOBUS PALLIDUS OPS<br>0151 EX CEREB MENINGEAL LES<br>0152 HEMISPHERECTOMY<br>0153 BRAIN LOBECTOMY<br>0159 OTHER BRAIN EXCISION	0201 LINEAR CRANIECTOMY<br>0202 ELEVATE SKULL FX FRAGMNT<br>0203 SKULL FLAP FORMATION<br>0204 BONE GRAFT TO SKULL<br>0205 SKULL PLATE INSERTION<br>0206 CRANIAL OSTEOPLASTY NEC<br>0207 SKULL PLATE REMOVAL<br>0211 SIMPLE SUTURE OF DURA<br>0212 BRAIN MENINGE REPAIR NEC<br>0213 MENINGE VESSEL LIGATION<br>0214 CHOROID PLEXECTOMY<br>022 VENTRICULOSTOMY<br>0231 VENTRICL SHUNT-HEAD/NECK<br>0232 VENTRI SHUNT-CIRCULA SYS<br>0233 VENTRICL SHUNT-THORAX<br>0234 VENTRICL SHUNT-ABDOMEN<br>0235 VENTRI SHUNT-UNINARY SYS<br>0239 OTHER VENTRICULAR SHUNT<br>0242 REPLACE VENTRICLE SHUNT<br>0243 REMOVE VENTRICLE SHUNT<br>0291 LYSIS CORTICAL ADHESION<br>0292 BRAIN REPAIR<br>0293 IMPLANT BRAIN STIMULATOR<br>0294 INSERT/REPLAC SKULL TONG<br>0299 SKULL & BRAIN OP NEC<br>0301 REMOVAL FB SPINAL CANAL<br>0302 REOPEN LAMINECTOMY SITE<br>0309 SPINAL CANAL EXPLOR NEC<br>031 INTRASPIN NERVE ROOT DIV<br>0321 PERCUTANEOUS CHORDOTOMY<br>0329 OTHER CHORDOTOMY<br>0332 SPINAL CORD/MENINGES BX<br>0339 OTHER SPINAL DX PROC<br>034 EXCIS SPINAL CORD LESION<br>0351 SPINE MENINGOCELE REPAIR<br>0352 MYELOMENINGOCEL REPAIR<br>0353 VERTEBRAL FX REPAIR<br>0359 SPINAL STRUCT REPAIR NEC<br>036 SPINAL CORD ADHESIOLYSIS<br>0371 SUBARACH-PERITON SHUNT<br>0372 SUBARACH-URETERAL SHUNT<br>0379 OTH SPINAL THECAL SHUNT<br>0393 INSERT SPINAL STIMULATOR<br>0394 REMOVE SPINAL STIMULATOR<br>0397 REVISE SPINE THECA SHUNT<br>0398 REMOVE SPINE THECA SHUNT<br>0399 SPINE CANAL STRUC OP NEC<br>0401 EXCISION ACOUSTC NEUROMA<br>0402 TRIGEMINAL NERV DIVISION

016 EXCISE SKULL LESION	0403 PERIPH NERVE DIV NEC
0404 PERIPH NERVE INCIS NEC	0681 TOTAL PARATHYROIDECTOMY
0405 GASSERIAN GANGLIONECTOMY	0689 OTHER PARATHYROIDECTOMY
0406 PERIPH GANGLIONECT NEC	0691 THYROID ISTHMUS DIVISION
0407 PERIPH NERV EXCISION NEC	0692 THYROID VESSEL LIGATION
0412 OPEN PERIPH NERVE BIOPSY	0693 THYROID SUTURE
0419 PERIPH NERVE DX PROC NEC	0694 THYROID REIMPLANTATION
043 PERIPHERAL NERVE SUTURE	0695 PARATHYROID REIMPLANT
0441 DECOMPRESS TRIGEM ROOT	0698 OTHER THYROID OPERATIONS
0442 CRAN NERV ROOT DECOM NEC	0699 OTHER PARATHYROID OPS
0443 CARPAL TUNNEL RELEASE	0700 ADRENAL EXPLORATION NOS
0444 TARSAL TUNNEL RELEASE	0701 UNILAT ADRENAL EXPLORAT
0449 PER NERVE ADHESIOLYS NEC	0702 BILAT ADRENAL EXPLORAT
045 PERIPHERAL NERVE GRAFT	0712 OPEN ADRENAL GLAND BX
046 PERIPH NERVE TRANSPOSIT	0713 TRANSFRONT PITUITARY BX
0471 HYPOGLOSS-FACIAL ANASTOM	0714 TRANSPHEN PITUITARY BX
0472 ACCESSORY-FACIAL ANASTOM	0715 PITUITARY BIOPSY NOS
0473 ACCESS-HYPOGLOSS ANASTOM	0716 THYMUS BIOPSY
0474 PERIPH NERV ANASTOM NEC	0717 PINEAL BIOPSY
0475 POSTOP REVIS PER NERV OP	0719 ENDOCRINE DX PROC NEC
0476 LATE REPAIR PER NERV INJ	0721 ADRENAL LESION EXCISION
0479 OTHER NEUROPLASTY	0722 UNILATERAL ADRENALECTOMY
0491 NEURECTASIS	0729 PART ADRENALECTOMY NEC
0492 IMPLANT PERIPH STIMULAT	073 BILATERAL ADRENALECTOMY
0493 REMOVE PERIPH STIMULATOR	0741 ADRENAL INCISION
0499 PERIPHERAL NERVE OPS NEC	0742 ADRENAL NERVE DIVISION
050 SYMPATH NERVE DIVISION	0743 ADRENAL VESSEL LIGATION
0511 SYMPATHETIC NERVE BIOPSY	0744 ADRENAL REPAIR
0519 SYMPATH NRV DX PROC NEC	0745 ADRENAL REIMPLANTATION
0521 SPHENOPALATIN GANGLIONEC	0749 ADRENAL OPERATION NEC
0522 CERVICAL SYMPATHECTOMY	0751 PINEAL FIELD EXPLORATION
0523 LUMBAR SYMPATHECTOMY	0752 PINEAL GLAND INCISION
0524 PRESACRAL SYMPATHECTOMY	0753 PARTIAL PINEALECTOMY
0525 PERIART SYMPATHECTOMY	0754 TOTAL PINEALECTOMY
0529 OTHER SYMPATHECTOMY	0759 PINEAL OPERATION NEC
0581 SYMPATHETIC NERVE REPAIR	0761 EXC PITUIT LES-TRANSFRON
0589 SYMPATHETIC NERVE OP NEC	0762 EXC PITUIT LES-TRANSPHEN
059 OTHER NERVOUS SYSTEM OPS	0763 PART EXCIS PITUITARY NOS
0602 REOPEN THYROID FIELD WND	0764 TOT EXC PITUIT-TRANSFRON
0609 INCIS THYROID FIELD NEC	0765 TOT EXC PITUIT-TRANSPHEN
0612 OPEN THYROID GLAND BX	0768 TOTAL EXC PITUITARY NEC
0613 PARATHYROID BIOPSY	0769 TOTAL EXC PITUITARY NOS
0619 THYR/PARATHY DX PROC NEC	0771 PITUITARY FOSSA EXPLORAT
062 UNILAT THYROID LOBECTOMY	0772 PITUITARY GLAND INCISION
0631 EXCISION THYROID LESION	0779 PITUITARY OPERATION NEC
0639 PART THYROIDECTOMY NEC	0780 THYMECTOMY NOS
064 COMPLETE THYROIDECTOMY	0781 PART EXCISION OF THYMUS
0650 SUBSTERN THYROIDECT NOS	0782 TOTAL EXCISION OF THYMUS
0651 PART SUBSTERN THYROIDECT	0791 THYMUS FIELD EXPLORATION
0652 TOT SUBSTERN THYROIDECT	0792 INCISION OF THYMUS
066 LINGUAL THYROID EXCISION	0793 REPAIR OF THYMUS

067 THYROGLOSS DUCT EXCISION
0799 THYMUS OPERATION NEC
0811 EYELID BIOPSY
0820 REMOVE EYELID LESION NOS
0821 CHALAZION EXCISION
0822 EXCISE MINOR LES LID NEC
0823 EXC MAJ LES LID PRT-THIC
0824 EXC MAJ LES LID FUL-THIC
0825 DESTRUCTION LID LESION
0831 PTOSIS REP-FRONT MUS SUT
0832 PTOSIS REP-FRON MUS SLNG
0833 PTOSIS REP-LEVAT MUS ADV
0834 PTOSIS REP-LEVAT MUS NEC
0835 PTOS REP-TARSAL TECHNIQ
0836 BLEPHAROPTOS REPAIR NEC
0837 REDUC OVERCORRECT PTOSIS
0838 CORRECT LID RETRACTION
0841 THERMOCAUT/ENTROPION REP
0842 SUTURE ENTROPION REPAIR
0843 WEDG RESEC ENTROPION REP
0844 LID RECONS ENTROPION REP
0849 ENTROPION/ECTROP REP NEC
0851 CANTHOTOMY
0852 BLEPHARORRHAPHY
0859 ADJUST LID POSITION NEC
0861 LID RECONST W SKIN GRAFT
0862 LID RECONST W MUC GRAFT
0863 LID RECONST W HAIR GRAFT
0864 LID RECON-TARSOCONJ FLAP
0869 LID RECONSTR W GRAFT NEC
0870 LID RECONSTRUCTION NOS
0871 LID MARG RECON-PART THIC
0872 LID RECONS-PART THIC NEC
0873 LID MARG RECONS FUL THIC
0874 LID RECONST-FUL THIC NEC
0891 ELECTROSURG LID EPILAT
0892 CRYOSURG LID EPILATION
0893 EYELID EPILATION NEC
0899 EYELID OPERATION NEC
090 LACRIMAL GLAND INCISION
0911 LACRIMAL GLAND BIOPSY
0912 LACRIMAL SAC BIOPSY
0919 LACRIMAL SYS DX PROC NEC
0920 EXC LACRIMAL GLAND NOS
0921 EXCIS LES LACRIMAL GLAND
0922 PART DACRYOADENECT NEC
0923 TOTAL DACRYOADENECTOMY
093 OTHER LACRIMAL GLAND OPS
0941 LACRIMAL PUNCTUM PROBE
0942 LAC CANALICULI PROBE
0943 NASOLACRIMAL DUCT PROBE
0794 THYMUS TRANSPLANTATION
0949 LAC PASSAGE MANIP NEC
0951 LAC PUNCTUM INCISION
0952 LAC CANALICULI INCISION
0953 LACRIMAL SAC INCISION
0959 LACRIM PASSAGE INCIS NEC
096 LACRIM SAC/PASSAGE EXCIS
0971 CORRECT EVERTED PUNCTUM
0972 PUNCTUM REPAIR NEC
0973 CANALICULUS REPAIR
0981 DACRYOCYSTORHINOSTOMY
0982 CONJUNCTIVOCYSTORHINOST
0983 CONJUNCTIVORHINOS W TUBE
0991 LAC PUNCTUM OBLITERATION
0999 LACRIMAL SYSTEM OP NEC
100 INCISE/REMOV CONJUNCT FB
101 CONJUNCTIVA INCISION NEC
1021 CONJUNCTIVAL BIOPSY
1029 CONJUNCTIVA DX PROC NEC
1031 EXCISE CONJUNCTIV LESION
1032 DESTRUCT CONJUNC LES NEC
1033 OTH CONJUNC DESTRUC PROC
1041 SYMBLEPH REP W FREE GRFT
1042 GRAFT CONJUNC CUL-DE-SAC
1043 CONJUN CUL-DE-SAC RX NEC
1044 CONJUNC FREE GRAFT NEC
1049 CONJUNCTIVOPLASTY NEC
105 CONJUNC/LID ADHESIOLYSIS
106 REPAIR CONJUNCT LACERAT
1091 SUBCONJUNCTIVAL INJECT
1099 CONJUNCTIVAL OP NEC
110 MAGNET REMOVAL CORNEA FB
111 CORNEAL INCISION
1121 CORNEAL SCRAPE FOR SMEAR
1122 CORNEAL BIOPSY
1129 CORNEAL DX PROC NEC
1131 PTERYGIUM TRANSPOSITION
1132 PTERYG EXC W CORNEA GRFT
1139 PTERYGIUM EXCISION NEC
1141 MECH REMOV CORNEA EPITH
1142 THERMOCAUT CORNEA LESION
1143 CRYOTHERAP CORNEA LESION
1149 DESTRUCT CORNEA LES NEC
1151 SUTURE CORNEA LACERATION
1152 REP CORNEA POSTOP DEHISC
1153 RX CORNEA LAC W CONJ FLP
1159 CORNEAL REPAIR NEC
1160 CORNEAL TRANSPLANT NOS
1161 LAM KERATPLAST W AUTGRFT
1162 LAMELLAR KERATOPLAST NEC
1163 PERF KERATOPL W AUTOGRFT

0944 NASOLAC DUCT INTUBAT<br>1169 CORNEAL TRANSPLANT NEC<br>1171 KERATOMILEUSIS<br>1172 KERATOPHAKIA<br>1173 KERATOPROSTHESIS<br>1174 THERMOKERATOPLASTY<br>1175 RADIAL KERATOTOMY<br>1176 EPIKERATOPHAKIA<br>1179 CORNEA RECONSTRUCT NEC<br>1191 CORNEAL TATTOOING<br>1192 REMOVE CORNEAL IMPLANT<br>1199 CORNEAL OPERATION NEC<br>1200 REMOV ANT SEGMNT FB NOS<br>1201 MAGNET REMOV ANT SEG FB<br>1202 NONMAG REMOV ANT SEG FB<br>1211 IRIDOTOMY W TRANSFIXION<br>1212 IRIDOTOMY NEC<br>1213 PROLAPSED IRIS EXCISION<br>1214 IRIDECTOMY NEC<br>1221 DX ASPIRAT-ANT CHAMBER<br>1222 IRIS BIOPSY<br>1229 ANT SEGMENT DX PROC NEC<br>1231 GONIOSYNECHIAE LYSIS<br>1232 ANT SYNECHIA LYSIS NEC<br>1233 POST SYNECHIAE LYSIS<br>1234 CORNEOVITREAL ADHESIOLYS<br>1235 COREOPLASTY<br>1239 IRIDOPLASTY NEC<br>1240 REMOV ANT SEGMNT LES NOS<br>1241 NONEXC DESTRUC IRIS LES<br>1242 EXCISION OF IRIS LESION<br>1243 NONEXC DESTR CIL BOD LES<br>1244 EXCISE CILIARY BODY LES<br>1251 GONIOPUNCTURE<br>1252 GONIOTOMY<br>1253 GONIOTOMY W GONIOPUNCTUR<br>1254 TRABECULOTOMY AB EXTERNO<br>1255 CYCLODIALYSIS<br>1259 FACILIT INTRAOC CIRC NEC<br>1261 TREPHIN SCLERA W IRIDECT<br>1262 THERMCAUT SCLER W IRIDEC<br>1263 IRIDENCLEISIS/IRIDOTASIS<br>1264 TRABECULECTOM AB EXTERNO<br>1265 SCLER FISTULIZ W IRIDECT<br>1266 POSTOP REVIS SCL FISTUL<br>1269 SCLER FISTULIZING OP NEC<br>1271 CYCLODIATHERMY<br>1272 CYCLOCRYOTHERAPY<br>1273 CYCLOPHOTOCOAGULATION<br>1274 CIL BODY DIMINUTION NOS<br>1279 GLAUCOMA PROCEDURE NEC	1164 PERFORAT KERATOPLAST NEC<br>1282 SCLERAL FISTULA REPAIR<br>1283 REVIS ANT SEG OP WND NEC<br>1284 DESTRUCT SCLERAL LESION<br>1285 REPAIR STAPHYLOM W GRAFT<br>1286 REP SCLER STAPHYLOMA NEC<br>1287 GRAFT REINFORCE SCLERA<br>1288 SCLERA REINFORCEMENT NEC<br>1289 SCLERAL OPERATION NEC<br>1291 THERAPEUT EVAC ANT CHAMB<br>1292 ANTERIOR CHAMBER INJECT<br>1293 REMOV EPITHEL DOWNGROWTH<br>1297 IRIS OPERATION NEC<br>1298 CILIARY BODY OP NEC<br>1299 ANTERIOR CHAMBER OP NEC<br>1300 REMOVE FB LENS NOS<br>1301 MAGNET REMOVE FB LENS<br>1302 NONMAGNET REMOVE FB LENS<br>1311 TEMP-INF INTRCAP LENS EX<br>1319 INTRACAPSUL LENS EXT NEC<br>132 LINEAR EXTRACAP LENS EXT<br>133 SIMPL ASPIR LENS EXTRACT<br>1341 CATARAC PHACOEMULS/ASPIR<br>1342 POST CATARAC FRAG/ASPIR<br>1343 CATARACT FRAG/ASPIR NEC<br>1351 TEMP-INF XTRACAP LENS EX<br>1359 EXTRACAP LENS EXTRAC NEC<br>1361 EXTRACAP LENS EXTRAC NEC<br>1362 EXTRACAP LENS EXTRAC NEC<br>1363 EXTRACAP LENS EXTRAC NEC<br>1364 AFTER-CATAR DISCISSION<br>1365 AFTER-CATARACT EXCISION<br>1366 AFTER CATAR FRAGMNTATION<br>1369 CATARACT EXTRACTION NEC<br>1370 INSERT PSEUDOPHAKOS NOS<br>1371 INSERT LENS AT CATAR EXT<br>1372 SECONDARY INSERT LENS<br>138 IMPLANTED LENS REMOVAL<br>139 OTHER OPERATIONS ON LENS<br>1390 OPERATION ON LENS NEC OCT06-<br>1391 IMPL INTRAOC TELESC PROS OCT06<br>1400 REMOV POST SEGMNT FB NOS<br>1401 MAGNET REMOV POST SEG FB<br>1402 NONMAG REMOV POST SEG FB<br>1411 DIAGNOST VITREOUS ASPIR<br>1419 DX PROC POST SEG NEC<br>1421 CHORIORET LES DIATHERMY<br>1422 CHORIORETIN LES CRYOTHER<br>1426 CHORIORET LES RADIOTHER<br>1427 CHORIORET LES RAD IMPLAN<br>1429 CHORIORET LES DESTR NEC

1281 SUTURE SCLERAL LACER	1431 RETINAL TEAR DIATHERMY
1432 RETINAL TEAR CRYOTHERAPY	1664 ENUC SOCKET REVIS NEC
1439 RETINAL TEAR REPAIR NEC	1665 2NDRY EXENT CAVITY GRAFT
1441 SCLERAL BUCKLE W IMPLANT	1666 REVIS EXENTER CAVITY NEC
1449 SCLERAL BUCKLING NEC	1669 2ND OP POST EYE REM NEC
1451 DETACH RETINA-DIATHERMY	1671 REMOVE OCULAR IMPLANT
1452 DETACH RETINA-CRYOTHERAP	1672 REMOVE ORBITAL IMPLANT
1453 DETACH RETINA XENON COAG	1681 REPAIR OF ORBITAL WOUND
1454 DETACH RETINA LASER COAG	1682 REPAIR EYEBALL RUPTURE
1455 DETACH RET PHOTOCOAG NOS	1689 EYE/ORBIT INJ REPAIR NEC
1459 REPAIR RETINA DETACH NEC	1692 EXCISION ORBITAL LESION
146 REMOV PROS MAT POST SEG	1693 EXCISION EYE LESION NOS
1471 ANTERIOR REMOV VITREOUS	1698 OPERATION ON ORBIT NEC
1472 VITREOUS REMOVAL NEC	1699 OPERATION ON EYEBALL NEC
1473 ANTERIOR MECHAN VITRECT	1821 PREAURICULAR SINUS EXCIS
1474 MECH VITRECTOMY NEC	1831 RAD EXCIS EXT EAR LES
1475 VITREOUS SUBSTITUT INJEC	1839 EXCIS EXTERNAL EAR NEC
1479 VITREOUS OPERATION NEC	185 CORRECTION PROMINENT EAR
149 OTHER POST SEGMENT OPS	186 EXT AUDIT CANAL RECONSTR
1501 EXTRAOC MUSC-TEND BIOPSY	1871 CONSTRUCTION EAR AURICLE
1509 EXTRAOC MUSC DX PROC NEC	1872 REATTACH AMPUTATED EAR
1511 ONE EXTRAOC MUS RECESS	1879 PLASTIC REP EXT EAR NEC
1512 1 EXTRAOC MUSCL ADVANCE	189 OTHER EXT EAR OPERATIONS
1513 1 EXTRAOC MUSCL RESECT	190 STAPES MOBILIZATION
1519 XTRAOC MUS OP/DETACH NEC	1911 STAPEDECT W REPLAC INCUS
1521 LENGTHEN 1 EXTRAOC MUSC	1919 STAPEDECTOMY NEC
1522 SHORTEN 1 EXTRAOC MUSC	1921 REV STAPDEC W INCUS REPL
1529 OP ON 1 EXTRAOC MUSC NEC	1929 STAPEDECTOMY REVIS NEC
153 TEMP DETACH >1 XTROC MUS	193 OSSICULAR CHAIN OP NEC
154 OTH OP ON >L EXTRAOC MUS	194 MYRINGOPLASTY
155 EXTRAOCUL MUS TRANSPOSIT	1952 TYPE 2 TYMPANOPLASTY
156 REVIS EXTRAOC MUSC SURG	1953 TYPE 3 TYMPANOPLASTY
157 EXTRAOC MUSC INJ REPAIR	1954 TYPE 4 TYMPANOPLASTY
159 OTH EXTRAOC MUS-TEND OP	1955 TYPE 5 TYMPANOPLASTY
1601 ORBITOTOMY W BONE FLAP	196 TYMPANOPLASTY REVISION
1602 ORBITOTOMY W IMPLANT	199 MIDDLE EAR REPAIR NEC
1609 ORBITOTOMY NEC	2001 MYRINGOTOMY W INTUBATION
161 REMOVE PENETRAT FB EYE	2021 MASTOID INCISION
1622 DIAGNOSTIC ASP OF ORBIT	2022 PETRUS PYRAM AIR CEL INC
1623 EYEBALL & ORBIT BIOPSY	2023 MIDDLE EAR INCISION
1629 EYEBAL/ORBIT DX PROC NEC	2032 MID & INNER EAR BIOPSY
1631 EYE EVISC W SYNCH IMPLAN	2039 MID/IN EAR DX PROC NEC
1639 EYEBALL EVISCERATION NEC	2041 SIMPLE MASTOIDECTOMY
1641 EYE ENUC/IMPLAN/MUSC ATT	2042 RADICAL MASTOIDECTOMY
1642 EYE ENUC W IMPLANT NEC	2049 MASTOIDECTOMY NEC
1649 EYEBALL ENUCLEATION NEC	2051 EXCISE MIDDLE EAR LESION
1651 RADICAL ORBITOMAXILLECT	2059 MIDDLE EAR EXCISION NEC
1652 ORBIT EXENT W BONE REMOV	2061 INNER EAR FENESTRATION
1659 ORBITAL EXENTERATION NEC	2062 REVIS INNER EAR FENESTRA
1661 2NDRY OCULAR IMP INSERT	2071 ENDOLYMPHATIC SHUNT
1662 REVIS/REINSERT OCUL IMP	2072 INNER EAR INJECTION

1663 REVIS ENUC SOCKET W GRFT	2079 INC/EXC/DESTR IN EAR NEC
2091 TYMPANOSYMPATHECTOMY	253 COMPLETE GLOSSECTOMY
2092 MASTOIDECTOMY REVISION	254 RADICAL GLOSSECTOMY
2093 REPAIR OVAL/ROUND WINDOW	2559 REPAIR OF TONGUE NEC
2095 ELECMAG HEAR DEV IMPLANT	2594 OTHER GLOSSOTOMY
2096 IMPLT COCHLEAR PROST NOS	2599 TONGUE OPERATION NEC
2097 IMP/REP SCHAN COCH PROS	2612 OPEN BX SALIV GLAND/DUCT
2098 IMP/REP MCHAN COCHL PROS	2621 SALIVARY CYST MARSUPIAL
2099 MID-INNER EAR OPS NEC	2629 SALIV LESION EXCIS NEC
2104 ETHMOID ART LIGAT-EPIST	2630 SIALOADENECTOMY NOS
2105 MAX ART LIG FOR EPISTAX	2631 PARTIAL SIALOADENECTOMY
2106 EXT CAROT ART LIG-EPIST	2632 COMPLETE SIALOADENECTOMY
2107 NASAL SEPT GRFT-EPISTAX	2641 SUTURE OF SALIV GLND LAC
2109 EPISTAXIS CONTROL NEC	2642 SALIVARY FISTULA CLOSURE
214 RESECTION OF NOSE	2649 SALIVARY REPAIR NEC
215 SUBMUC NASAL SEPT RESECT	2699 SALIVARY OPERATION NEC
2161 DIATHER/CRYO TURBINECTOM	270 DRAIN FACE & MOUTH FLOOR
2162 TURBINATE FRACTURE	271 INCISION OF PALATE
2169 TURBINECTOMY NEC	2721 BONY PALATE BIOPSY
2172 OPEN REDUCTION NASAL FX	2722 UVULA AND SOFT PALATE BX
2182 NASAL FISTULA CLOSURE	2731 LOC EXC BONY PALATE LES
2183 TOT NASAL RECONSTRUCTION	2732 WIDE EXC BONY PALATE LES
2184 REVISION RHINOPLASTY	2742 WIDE EXCISION OF LIP LES
2185 AUGMENTATION RHINOPLASTY	2743 EXCISION OF LIP LES NEC
2186 LIMITED RHINOPLASTY	2749 EXCISION OF MOUTH NEC
2187 RHINOPLASTY NEC	2753 CLOSURE OF MOUTH FISTULA
2188 SEPTOPLASTY NEC	2754 REPAIR OF CLEFT LIP
2189 NASAL REPAIR NEC	2755 FULL-THICK GRFT TO MOUTH
2199 NASAL OPERATION NEC	2756 SKIN GRAFT TO MOUTH NEC
2212 OPEN BIOPSY NASAL SINUS	2757 PEDICLE ATTACH TO MOUTH
2231 RADICAL MAXILLARY ANTROT	2759 MOUTH REPAIR NEC
2239 EXT MAXILLARY ANTROT NEC	2761 SUTURE OF PALATE LACERAT
2241 FRONTAL SINUSOTOMY	2762 CLEFT PALATE CORRECTION
2242 FRONTAL SINUSECTOMY	2763 REVIS CLEFT PALAT REPAIR
2250 SINUSOTOMY NOS	2769 OTH PLASTIC REPAIR PALAT
2251 ETHMOIDOTOMY	2771 INCISION OF UVULA
2252 SPHENOIDOTOMY	2772 EXCISION OF UVULA
2253 MULTIPLE SINUS INCISION	2773 REPAIR OF UVULA
2260 SINUSECTOMY NOS	2779 OTHER UVULA OPERATIONS
2261 C-LUC EXC MAX SINUS LES	2792 MOUTH INCISION NOS
2262 EXC MAX SINUS LESION NEC	2799 ORAL CAVITY OPS NEC
2263 ETHMOIDECTOMY	280 PERITONSILLAR I & D
2264 SPHENOIDECTOMY	2811 TONSIL&ADENOID BIOPSY
2271 NASAL SINUS FISTULA CLOS	2819 TONSIL&ADENOID DX OP NEC
2279 NASAL SINUS REPAIR NEC	282 TONSILLECTOMY
229 OTHER NASAL SINUS OPS	283 TONSILLECTOMY/ADENOIDEC
242 GINGIVOPLASTY	284 EXCISION OF TONSIL TAG
244 EXC OF DENTAL LES OF JAW	285 EXCISION LINGUAL TONSIL
245 ALVEOLOPLASTY	286 ADENOIDECTOMY
2502 OPEN BIOPSY OF TONGUE	287 HEMORR CONTRL POST T & A
251 DESTRUCTION TONGUE LES	2891 INCIS TO REMOV TONSIL FB

252 PARTIAL GLOSSECTOMY	2892 EXCIS TONSIL/ADENOID LES
2899 TONSIL/ADENOID OPS NEC	3225 THOR ABLTN LUNG LES/TISS OCT06
290 PHARYNGOTOMY	3226 ABLTN LUNG TISS NEC/NOS OCT06-
292 EXC BRANCHIAL CLEFT CYST	3229 DESTROY LOC LUNG LES NEC
293 EXC BRANCHIAL CLEFT CYST	323 SEGMENTAL LUNG RESECTION
2931 CRICOPHARYNGEAL MYOTOMY	324 LOBECTOMY OF LUNG
2932 PHARYNGEAL DIVERTICULEC	325 COMPLETE PNEUMONECTOMY
2933 PHARYNGECTOMY	326 RAD DISSEC THORAC STRUCT
2939 EXCIS/DESTR LES PHAR NEC	329 OTHER EXCISION OF LUNG
294 PLASTIC OP ON PHARYNX	330 INCISION OF BRONCHUS
2951 SUTURE OF PHARYNGEAL LAC	331 INCISION OF LUNG
2952 CLOS BRANCH CLEFT FISTUL	3325 OPEN BRONCHIAL BIOPSY
2953 CLOS PHARYNX FISTULA NEC	3327 CLOS ENDOSCOPIC LUNG BX
2954 LYSIS PHARYNGEAL ADHES	3328 OPEN LUNG BIOPSY
2959 PHARYNGEAL REPAIR NEC	3329 BRONCH/LUNG DX PROC NEC
2992 DIVIS GLOSSOPHARYNG NERV	3334 THORACOPLASTY
2999 PHARYNGEAL OPERATION NEC	3339 SURG COLLAPS OF LUNG NEC
3001 LARYNX CYST MARSUPIALIZ	3341 BRONCHIAL LACERAT SUTURE
3009 DESTRUCT LARYNX LES NEC	3342 BRONCHIAL FISTULA CLOS
301 HEMILARYNGECTOMY	3343 LUNG LACERATION CLOSURE
3021 EPIGLOTTIDECTOMY	3348 BRONCHIAL REPAIR NEC
3022 VOCAL CORDECTOMY	3349 LUNG REPAIR NEC
3029 OTHER PART LARYNGECTOMY	335 LUNG REPAIR NEC
303 COMPLETE LARYNGECTOMY	3350 LUNG TRANSPLANT NOS
304 RADICAL LARYNGECTOMY	3351 UNILAT LUNG TRANSPLANT
3121 MEDIASTINAL TRACHEOSTOMY	3352 BILAT LUNG TRANSPLANT
3129 OTHER PERM TRACHEOSTOMY	336 COMB HEART/LUNG TRANSPLA
313 INCIS LARYNX TRACHEA NEC	3392 BRONCHIAL LIGATION
3145 OPN BX LARYNX OR TRACHEA	3393 PUNCTURE OF LUNG
315 LOCAL DESTRUC TRACH LES	3398 BRONCHIAL OPERATION NEC
3161 SUTURE OF LARYNGEAL LAC	3399 LUNG OPERATION NEC
3162 LARYNGEAL FISTULA CLOS	3402 EXPLORATORY THORACOTOMY
3163 LARYNGOSTOMY REVISION	3403 REOPEN THORACOTOMY SITE
3164 LARYNGEAL FX REPAIR	341 INCISION OF MEDIASTINUM
3169 OTHER LARYNGEAL REPAIR	3421 TRANSPLEURA THORACOSCOPY
3171 SUTURE OF TRACHEAL LACER	3422 MEDIASTINOSCOPY
3172 CLOSURE OF TRACHEOSTOMY	3426 OPEN MEDIASTINAL BIOPSY
3173 TRACHEA FISTULA CLOS NEC	3427 BIOPSY OF DIAPHRAGM
3174 REVISION OF TRACHEOSTOMY	3428 DX PROCEDURE THORAX NEC
3175 TRACHEAL RECONSTRUCTION	3429 DX PROC MEDIASTINUM NEC
3179 OTHER TRACHEAL REPAIR	343 DESTRUCT MEDIASTIN LES
3191 LARYNGEAL NERV DIVISION	344 DESTRUCT CHEST WALL LES
3192 LYSIS TRACH/LARYNX ADHES	3451 DECORTICATION OF LUNG
3198 OTH LARYNGEAL OPERATION	3459 OTHER PLEURAL EXCISION
3199 OTHER TRACHEAL OPERATION	346 SCARIFICATION OF PLEURA
320 OTHER TRACHEAL OPERATION	3473 CLOS THORACIC FISTUL NEC
3209 OTHER DESTRUC BRONC LES	3474 PECTUS DEFORMITY REPAIR
321 OTHER BRONCHIAL EXCISION	3479 OTHER CHEST WALL REPAIR
3221 EMPHYSEMA BLEB PLICATION	3481 EXCISE DIAPHRAGM LESION
3222 LUNG VOL REDUCTION SURG	3482 SUTURE DIAPHRAGM LACERAT
3223 OPEN ABLTN LUNG LES/TISS OCT06-	3483 CLOSE DIAPHRAGM FISTULA

3224 PERC ABLTN LUNG LES/TISS OCT06-<br>3485 IMPLANT DIAPHRA PACEMAKE<br>3489 DIAPHRAGM OPERATION NEC<br>3493 REPAIR OF PLEURA<br>3499 THORACIC OPERATION NEC<br>3500 CLOSED VALVOTOMY NOS<br>3501 CLOSED AORTIC VALVOTOMY<br>3502 CLOSED MITRAL VALVOTOMY<br>3503 CLOSED PULMON VALVOTOMY<br>3504 CLOSED TRICUSP VALVOTOMY<br>3510 OPEN VALVULOPLASTY NOS<br>3511 OPN AORTIC VALVULOPLASTY<br>3512 OPN MITRAL VALVULOPLASTY<br>3513 OPN PULMON VALVULOPLASTY<br>3514 OPN TRICUS VALVULOPLASTY<br>3520 REPLACE HEART VALVE NOS<br>3521 REPLACE AORT VALV-TISSUE<br>3522 REPLACE AORTIC VALVE NEC<br>3523 REPLACE MITR VALV-TISSUE<br>3524 REPLACE MITRAL VALVE NEC<br>3525 REPLACE PULM VALV-TISSUE<br>3526 REPLACE PULMON VALVE NEC<br>3527 REPLACE TRIC VALV-TISSUE<br>3528 REPLACE TRICUSP VALV NEC<br>3531 PAPILLARY MUSCLE OPS<br>3532 CHORDAE TENDINEAE OPS<br>3533 ANNULOPLASTY<br>3534 INFUNDIBULECTOMY<br>3535 TRABECUL CARNEAE CORD OP<br>3539 TISS ADJ TO VALV OPS NEC<br>3542 CREATE SEPTAL DEFECT<br>3550 PROSTH REP HRT SEPTA NOS<br>3551 PROS REP ATRIAL DEF-OPN<br>3552 PROS REPAIR ATRIA DEF-CL<br>3553 PROST REPAIR VENTRIC DEF<br>3554 PROS REP ENDOCAR CUSHION<br>3555 PROS REP VENTRC DEF-CLOS OCT06-<br>3560 GRFT REPAIR HRT SEPT NOS<br>3561 GRAFT REPAIR ATRIAL DEF<br>3562 GRAFT REPAIR VENTRIC DEF<br>3563 GRFT REP ENDOCAR CUSHION<br>3570 HEART SEPTA REPAIR NOS<br>3571 ATRIA SEPTA DEF REP NEC<br>3572 VENTR SEPTA DEF REP NEC<br>3573 ENDOCAR CUSHION REP NEC<br>3581 TOT REPAIR TETRAL FALLOT<br>3582 TOTAL REPAIR OF TAPVC<br>3583 TOT REP TRUNCUS ARTERIOS<br>3584 TOT COR TRANSPOS GRT VES<br>3591 INTERAT VEN RETRN TRANSP<br>3592 CONDUIT RT VENT-PUL ART	3484 OTHER DIAPHRAGM REPAIR<br>3594 CONDUIT ARTIUM-PULM ART<br>3595 HEART REPAIR REVISION<br>3596 PERC HEART VALVULOPLASTY<br>3598 OTHER HEART SEPTA OPS<br>3599 OTHER HEART VALVE OPS<br>3600 OTHER HEART VALVE OPS<br>3601 PTCA-1 VES/ATH W/O AGENT<br>3602 PTCA-1 VES/ATH W AGENT<br>3603 OPEN CORONRY ANGIOPLASTY<br>3605 PTCA-MULTIPLE VESSEL/ATH<br>3609 REM OF COR ART OBSTR NEC<br>3610 AORTOCORONARY BYPASS NOS<br>3611 AORTOCOR BYPAS-1 COR ART<br>3612 AORTOCOR BYPAS-2 COR ART<br>3613 AORTOCOR BYPAS-3 COR ART<br>3614 AORTCOR BYPAS-4+ COR ART<br>3615 1 INT MAM-COR ART BYPASS<br>3616 2 INT MAM-COR ART BYPASS<br>3617 ABD-CORON ARTERY BYPASS<br>3619 HRT REVAS BYPS ANAS NEC<br>362 ARTERIAL IMPLANT REVASC<br>363 ARTERIAL IMPLANT REVASC<br>3631 OPEN CHEST TRANS REVASC<br>3632 OTH TRANSMYO REVASCULAR<br>3633 ENDO TRANSMYO REVASCULAR OCT06-<br>3634 PERC TRANSMYO REVASCULAR OCT06-<br>3639 OTH HEART REVASCULAR<br>3691 CORON VESS ANEURYSM REP<br>3699 HEART VESSEL OP NEC<br>3710 INCISION OF HEART NOS<br>3711 CARDIOTOMY<br>3712 PERICARDIOTOMY<br>3724 PERICARDIAL BIOPSY<br>3731 PERICARDIECTOMY<br>3732 HEART ANEURYSM EXCISION<br>3733 EXC/DEST HRT LESION OPEN<br>3734 EXC/DEST HRT LES OTHER<br>3735 PARTIAL VENTRICULECTOMY<br>374 HEART & PERICARD REPAIR<br>3741 IMPL CARDIAC SUPPORT DEV OCT<br>3749 HEART/PERICARD REPR NEC OCT05-<br>375 HEART & PERICARD REPAIR<br>3751 HEART TRANSPLANTATION OCT03-<br>3752 IMPLANT TOT REP HRT SYS<br>3753 REPL/REP THORAC UNIT HRT<br>3754 REPL/REP OTH TOT HRT SYS<br>3761 PULSATION BALLOON IMPLAN<br>3762 IMPLANT HRT ASST SYS NEC

3593 CONDUIT LEFT VENTR-AORTA	3763 REPLACE HRT ASSIST SYST
3764 REMOVE HEART ASSIST SYS	3839 LEG VEIN RESECT/ANASTOM
3765 IMP EXT PUL HRT ASST SYS	3840 VESSEL RESECT/REPLAC NOS
3766 IMP IMP PUL HRT ASST SYS	3841 INTRACRAN VES RESEC-REPL
3767 IMP CARDIOMYOSTIMUL SYS	3842 HEAD/NECK VES RESEC-REPL
3774 INT OR REPL LEAD EPICAR	3843 ARM VES RESECT W REPLACE
3775 REVISION OF LEAD	3844 RESECT ABDM AORTA W REPL
3776 REPL TV ATRI-VENT LEAD	3845 RESECT THORAC VES W REPL
3777 REMOVAL OF LEAD W/O REPL	3846 ABD ARTERY RESEC W REPLA
3779 REVIS OR RELOCATE POCKET	3847 ABD VEIN RESECT W REPLAC
3780 INT OR REPL PERM PACEMKR	3848 LEG ARTERY RESEC W REPLA
3785 REPL PACEM W 1-CHAM, NON	3849 LEG VEIN RESECT W REPLAC
3786 REPL PACEM 1-CHAM, RATE	3850 VARICOSE V LIG-STRIP NOS
3787 REPL PACEM W DUAL-CHAM	3851 INTCRAN VAR V LIG-STRIP
3789 REVISE OR REMOVE PACEMAK	3852 HEAD/NECK VAR V LIG-STR
3790 INS LEFT ATR APPEND DEV (OCT 04)	3853 ARM VARICOSE V LIG-STRIP
3791 OPN CHEST CARDIAC MASSAG	3855 THORAC VAR V LIG-STRIP
3794 IMPLT/REPL CARDDEFIB TOT	3857 ABD VARICOS V LIGA-STRIP
3795 IMPLT CARDIODEFIB LEADS	3859 LEG VARICOS V LIGA-STRIP
3796 IMPLT CARDIODEFIB GENATR	3860 EXCISION OF VESSEL NOS
3797 REPL CARDIODEFIB LEADS	3861 INTRACRAN VESSEL EXCIS
3798 REPL CARDIODEFIB GENRATR	3862 HEAD/NECK VESSEL EXCIS
3799 OTHER HEART/PERICARD OPS	3863 ARM VESSEL EXCISION
3800 INCISION OF VESSEL NOS	3864 EXCISION OF AORTA
3801 INTRACRAN VESSEL INCIS	3865 THORACIC VESSEL EXCISION
3802 HEAD/NECK VES INCIS NEC	3866 ABDOMINAL ARTERY EXCIS
3803 UPPER LIMB VESSEL INCIS	3867 ABDOMINAL VEIN EXCISION
3804 INCISION OF AORTA	3868 LEG ARTERY EXCISION
3805 THORACIC VESSEL INC NEC	3869 LEG VEIN EXCISION
3806 ABDOMEN ARTERY INCISION	387 INTERRUPTION VENA CAVA
3807 ABDOMINAL VEIN INCISION	3880 SURG VESSEL OCCLUS NEC
3808 LOWER LIMB ARTERY INCIS	3881 OCCLUS INTRACRAN VES NEC
3809 LOWER LIMB VEIN INCISION	3882 OCCLUS HEAD/NECK VES NEC
3810 ENDARTERECTOMY NOS	3883 OCCLUDE ARM VESSEL NEC
3811 INTRACRAN ENDARTERECTOMY	3884 OCCLUDE AORTA NEC
3812 HEAD & NECK ENDARTER NEC	3885 OCCLUDE THORACIC VES NEC
3813 UPPER LIMB ENDARTERECTOM	3886 OCCLUDE ABD ARTERY NEC
3814 ENDARTERECTOMY OF AORTA	3887 OCCLUDE ABD VEIN NEC
3815 THORACIC ENDARTERECTOMY	3888 OCCLUDE LEG ARTERY NEC
3816 ABDOMINAL ENDARTERECTOMY	3889 OCCLUDE LEG VEIN NEC
3818 LOWER LIMB ENDARTERECT	390 SYSTEMIC-PULM ART SHUNT
3821 BLOOD VESSEL BIOPSY	391 INTRA-ABD VENOUS SHUNT
3829 BLOOD VESSEL DX PROC NEC	3921 CAVAL-PULMON ART ANASTOM
3830 VESSEL RESECT/ANAST NOS	3922 AORTA-SUBCLV-CAROT BYPAS
3831 INTRACRAN VES RESEC-ANAS	3923 INTRATHORACIC SHUNT NEC
3832 HEAD/NECK VES RESEC-ANAS	3924 AORTA-RENAL BYPASS
3833 ARM VESSEL RESECT/ANAST	3925 AORTA-ILIAC-FEMOR BYPASS
3834 AORTA RESECTION & ANAST	3926 INTRA-ABDOMIN SHUNT NEC
3835 THOR VESSEL RESECT/ANAST	3927 DIALYSIS ARTERIOVENOSTOM
3836 ABD VESSEL RESECT/ANAST	3928 EXTRACRAN-INTRACR BYPASS
3837 ABD VEIN RESECT & ANAST	3929 VASC SHUNT & BYPASS NEC

3838 LEG ARTERY RESECT/ANAST	3930 SUTURE OF VESSEL NOS
3931 SUTURE OF ARTERY	3839 LEG VEIN RESECT/ANASTOM
3932 SUTURE OF VEIN	3840 VESSEL RESECT/REPLAC NOS
3941 POSTOP VASC OP HEM CONTR	3841 INTRACRAN VES RESEC-REPL
3942 REVIS REN DIALYSIS SHUNT	3842 HEAD/NECK VES RESEC-REPL
3943 REMOV REN DIALYSIS SHUNT	3843 ARM VES RESECT W REPLACE
3949 VASC PROC REVISION NEC	3844 RESECT ABDM AORTA W REPL
3950 ANGIO/ATH NON-CORO VES	3845 RESECT THORAC VES W REPL
3951 CLIPPING OF ANEURYSM	3846 ABD ARTERY RESEC W REPLA
3952 ANEURYSM REPAIR NEC	3847 ABD VEIN RESECT W REPLAC
3953 ARTERIOVEN FISTULA REP	3848 LEG ARTERY RESEC W REPLA
3954 RE-ENTRY OPERATION	3849 LEG VEIN RESECT W REPLAC
3955 REIMPLAN ABERR RENAL VES	3850 VARICOSE V LIG-STRIP NOS
3956 REPAIR VESS W TIS PATCH	3851 INTCRAN VAR V LIG-STRIP
3957 REP VESS W SYNTH PATCH	3852 HEAD/NECK VAR V LIG-STR
3958 REPAIR VESS W PATCH NOS	3853 ARM VARICOSE V LIG-STRIP
3959 REPAIR OF VESSEL NEC	3855 THORAC VAR V LIG-STRIP
397 PER CARDIOPULMON BYPASS	3857 ABD VARICOS V LIGA-STRIP
3971 ENDO IMPL GRFT ABD AORTA	3859 LEG VARICOS V LIGA-STRIP
3972 ENDOVASC REPAIR HEAD VES	3860 EXCISION OF VESSEL NOS
3973 ENDO IMP GRFT THOR AORTA OCT05-	3861 INTRACRAN VESSEL EXCIS
3974 ENDO REM OBS HD/NECK VES OCT06-	3862 HEAD/NECK VESSEL EXCIS
3979 ENDO REPAIR OTHER VESSEL	3863 ARM VESSEL EXCISION
398 VASCULAR BODY OPERATIONS	3864 EXCISION OF AORTA
3991 FREEING OF VESSEL	3865 THORACIC VESSEL EXCISION
3992 VEIN INJECT-SCLEROS AGNT	3866 ABDOMINAL ARTERY EXCIS
3993 INSERT VES-TO-VES CANNUL	3867 ABDOMINAL VEIN EXCISION
3994 REPLAC VES-TO-VES CANNUL	3868 LEG ARTERY EXCISION
3998 HEMORRHAGE CONTROL NOS	3869 LEG VEIN EXCISION
3999 VESSEL OPERATION NEC	387 INTERRUPTION VENA CAVA
400 INCIS LYMPHATIC STRUCTUR	3880 SURG VESSEL OCCLUS NEC
4011 LYMPHATIC STRUCT BIOPSY	3881 OCCLUS INTRACRAN VES NEC
4019 LYMPHATIC DIAG PROC NEC	3882 OCCLUS HEAD/NECK VES NEC
4021 EXCIS DEEP CERVICAL NODE	3883 OCCLUDE ARM VESSEL NEC
4022 EXCISE INT MAMMARY NODE	3884 OCCLUDE AORTA NEC
4023 EXCISE AXILLARY NODE	3885 OCCLUDE THORACIC VES NEC
4024 EXCISE INGUINAL NODE	3886 OCCLUDE ABD ARTERY NEC
4029 SIMP EXC LYMPH STRUC NEC	3887 OCCLUDE ABD VEIN NEC
403 REGIONAL LYMPH NODE EXC	3888 OCCLUDE LEG ARTERY NEC
4040 RAD NECK DISSECTION NOS	3889 OCCLUDE LEG VEIN NEC
4041 UNILAT RAD NECK DISSECT	390 SYSTEMIC-PULM ART SHUNT
4042 BILAT RAD NECK DISSECT	391 INTRA-ABD VENOUS SHUNT
4050 RAD NODE DISSECTION NOS	3921 CAVAL-PULMON ART ANASTOM
4051 RAD DISSEC AXILLARY NODE	3922 AORTA-SUBCLV-CAROT BYPAS
4052 RAD DISSEC PERIAORT NODE	3923 INTRATHORACIC SHUNT NEC
4053 RAD DISSECT ILIAC NODES	3924 AORTA-RENAL BYPASS
4054 RADICAL GROIN DISSECTION	3925 AORTA-ILIAC-FEMOR BYPASS
4059 RAD NODE DISSECTION NEC	3926 INTRA-ABDOMIN SHUNT NEC
4061 THORAC DUCT CANNULATION	3927 DIALYSIS ARTERIOVENOSTOM
4062 THORACIC DUCT FISTULIZAT	3928 EXTRACRAN-INTRACR BYPASS
4063 CLOSE THORACIC DUCT FIST	3929 VASC SHUNT & BYPASS NEC

4064 LIGATE THORACIC DUCT	3930 SUTURE OF VESSEL NOS
4069 THORACIC DUCT OP NEC	4268 STERN INTERPOSITION NEC
409 LYMPH STRUCTURE OP NEC	4269 STERN ESOPHAG ANAST NEC
410 BONE MARROW TRNSPLNT	427 ESOPHAGOMYOTOMY
4100 BONE MARROW TRNSPLNT NOS	4282 SUTURE ESOPHAGEAL LACER
4101 AUTO BONE MT W/O PURG	4283 ESOPHAGOSTOMY CLOSURE
4102 ALO BONE MARROW TRNSPLNT	4284 ESOPH FISTULA REPAIR NEC
4103 ALLOGRFT BONE MARROW NOS	4285 ESOPHAG STRICTURE REPAIR
4104 AUTO HEM STEM CT W/O PUR	4286 PROD SUBQ TUNNEL NO ANAS
4105 ALLO HEM STEM CT W/O PUR	4287 ESOPHAGEAL GRAFT NEC
4106 CORD BLD STEM CELL TRANS	4289 ESOPHAGEAL REPAIR NEC
4107 AUTO HEM STEM CT W PURG	4291 LIGATION ESOPH VARIX
4108 ALLO HEM STEM CT W PURG	430 GASTROTOMY
4109 AUTO BONE MT W PURGING	431 GASTROTOMY
412 SPLENOTOMY	432 OTHER GASTROSTOMY
4133 OPEN SPLEEN BIOPSY	433 PYLOROMYOTOMY
4141 SPLENIC CYST MARSUPIAL	4342 LOCAL GASTR EXCISION NEC
4142 EXC SPLENIC LESION/TISS	4349 LOCAL GASTR DESTRUCT NEC
4143 PARTIAL SPLENECTOMY	435 PROXIMAL GASTRECTOMY
415 TOTAL SPLENECTOMY	436 DISTAL GASTRECTOMY
4193 EXC OF ACCESSORY SPLEEN	437 PART GASTREC W JEJ ANAST
4194 SPLEEN TRANSPLANTATION	4381 PART GAST W JEJ TRANSPOS
4195 REPAIR OF SPLEEN	4389 PARTIAL GASTRECTOMY NEC
4199 SPLEEN OPERATION NEC	4391 TOT GAST W INTES INTERPO
4201 ESOPHAGEAL WEB INCISION	4399 TOTAL GASTRECTOMY NEC
4209 ESOPHAGEAL INCISION NEC	4400 VAGOTOMY NOS
4210 ESOPHAGOSTOMY NOS	4401 TRUNCAL VAGOTOMY
4211 CERVICAL ESOPHAGOSTOMY	4402 HIGHLY SELECT VAGOTOMY
4212 ESOPH POUCH EXTERIORIZAT	4403 SELECTIVE VAGOTOMY NEC
4219 EXT FISTULIZAT ESOPH NEC	4411 TRANSABDOMIN GASTROSCOPY
4221 ESOPHAGOSCOPY BY INCIS	4415 OPEN GASTRIC BIOPSY
4225 OPEN BIOPSY OF ESOPHAGUS	442 GASTRIC DIAGNOS PROC NEC
4231 LOC EXCIS ESOPH DIVERTIC	4421 DILATE PYLORUS, INCISION
4232 LOCAL EXCIS ESOPHAG NEC	4429 OTHER PYLOROPLASTY
4239 DESTRUCT ESOPHAG LES NEC	4431 HIGH GASTRIC BYPASS
4240 ESOPHAGECTOMY NOS	4432 PERCU GASTROJEJUNOSTOMY
4241 PARTIAL ESOPHAGECTOMY	4438 LAP GASTROENTEROSTOMY (OCT
4242 TOTAL ESOPHAGECTOMY	4439 GASTROENTEROSTOMY NEC
4251 THORAC ESOPHAGOESOPHAGOS	4440 SUTURE PEPTIC ULCER NOS
4252 THORAC ESOPHAGOGASTROST	4441 SUT GASTRIC ULCER SITE
4253 THORAC SM BOWEL INTERPOS	4442 SUTURE DUODEN ULCER SITE
4254 THORAC ESOPHAGOENTER NEC	445 REVISION GASTRIC ANASTOM
4255 THORAC LG BOWEL INTERPOS	4461 SUTURE GASTRIC LACERAT
4256 THORAC ESOPHAGOCOLOS NEC	4463 CLOSE GASTRIC FISTUL NEC
4258 THORAC INTERPOSITION NEC	4464 GASTROPEXY
4259 THORAC ESOPHAG ANAST NEC	4465 ESOPHAGOGASTROPLASTY
4261 STERN ESOPHAGOESOPHAGOST	4466 CREAT ESOPHAGASTR SPHINC
4262 STERN ESOPHAGOGASTROSTOM	4467 LAP CREAT ESOPH SPHINCT (OCT
4263 STERN SM BOWEL INTERPOS	4468 LAPAROSCOP GASTROPLSTY (OCT
4264 STERN ESOPHAGOENTER NEC	4469 GASTRIC REPAIR NEC
4265 STERN LG BOWEL INTERPOS	4491 LIGATE GASTRIC VARICES

4266 STERN ESOPHAGOCOLOS NEC	4492 INTRAOP GASTRIC MANIPUL
4495 LAP GASTRIC RESTRIC PROC (OCT 04)	4640 INTEST STOMA REVIS NOS
4496 LAP REV GAST RESTRI PROC (OCT 04)	4641 SM BOWEL STOMA REVISION
4497 LAP REM GAST RESTRIC DEV (OCT 04)	4642 PERICOLOST HERNIA REPAIR
4498 ADJUST GAST RESTRICT DEV (OCT 04)	4643 LG BOWEL STOMA REVIS NEC
4499 GASTRIC OPERATION NEC	4650 INTEST STOMA CLOSURE NOS
4500 INTESTINAL INCISION NOS	4651 SM BOWEL STOMA CLOSURE
4501 DUODENAL INCISION	4652 LG BOWEL STOMA CLOSURE
4502 SMALL BOWEL INCISION NEC	4660 INTESTINAL FIXATION NOS
4503 LARGE BOWEL INCISION	4661 SM BOWEL-ABD WALL FIXAT
4511 TRANSAB SM BOWEL ENDOSC	4662 SMALL BOWEL FIXATION NEC
4515 OPEN SMALL BOWEL BIOPSY	4663 LG BOWEL-ABD WALL FIXAT
4521 TRANSAB LG BOWEL ENDOSC	4664 LARGE BOWEL FIXATION NEC
4526 OPEN LARGE BOWEL BIOPSY	4671 DUODENAL LACERAT SUTURE
4531 OTH EXCISE DUODENUM LES	4672 DUODENAL FISTULA CLOSURE
4532 DESTRUCT DUODEN LES NEC	4673 SMALL BOWEL SUTURE NEC
4533 LOCAL EXCIS SM BOWEL NEC	4674 CLOSE SM BOWEL FIST NEC
4534 DESTR SM BOWEL LES NEC	4675 SUTURE LG BOWEL LACERAT
4541 EXCISE LG INTESTINE LES	4676 CLOSE LG BOWEL FISTULA
4549 DESTRUC LG BOWEL LES NEC	4679 REPAIR OF INTESTINE NEC
4550 INTEST SEG ISOLAT NOS	4680 INTRA-AB BOWEL MANIP NOS
4551 SM BOWEL SEGMENT ISOLAT	4681 INTRA-ABD SM BOWEL MANIP
4552 LG BOWEL SEGMENT ISOLAT	4682 INTRA-ABD LG BOWEL MANIP
4561 MULT SEG SM BOWEL EXCIS	4691 MYOTOMY OF SIGMOID COLON
4562 PART SM BOWEL RESECT NEC	4692 MYOTOMY OF COLON NEC
4563 TOTAL REMOVAL SM BOWEL	4693 REVISE SM BOWEL ANASTOM
4571 MULT SEG LG BOWEL EXCIS	4694 REVISE LG BOWEL ANASTOM
4572 CECECTOMY	4697 TRANSPLANT OF INTESTINE
4573 RIGHT HEMICOLECTOMY	4699 INTESTINAL OP NEC
4574 TRANSVERSE COLON RESECT	470 INTESTINAL OP NEC
4575 LEFT HEMICOLECTOMY	4701 LAP APPENDECTOMY
4576 SIGMOIDECTOMY	4709 OTHER APPENDECTOMY
4579 PART LG BOWEL EXCIS NEC	471 OTHER APPENDECTOMY
458 TOT INTRA-ABD COLECTOMY	4711 LAP INCID APPENDECTOMY
4590 INTESTINAL ANASTOM NOS	4719 OTHER INCID APPENDECTOMY
4591 SM-TO-SM BOWEL ANASTOM	472 DRAIN APPENDICEAL ABSC
4592 SM BOWEL-RECT STUMP ANAS	4791 APPENDICOSTOMY
4593 SMALL-TO-LARGE BOWEL NEC	4792 CLOSE APPENDICEAL FISTUL
4594 LG-TO-LG BOWEL ANASTOM	4799 APPENDICEAL OPS NEC
4595 ANAL ANASTOMOSIS	480 PROCTOTOMY
4601 SM BOWEL EXTERIORIZATION	481 PROCTOSTOMY
4602 RESECT EXT SEG SM BOWEL	4821 TRANSAB PROCTOSIGMOIDOSC
4603 LG BOWEL EXTERIORIZATION	4825 OPEN RECTAL BIOPSY
4604 RESECT EXT SEG LG BOWEL	4835 LOCAL EXCIS RECTAL LES
4610 COLOSTOMY NOS	4841 SOAVE SUBMUC RECT RESECT
4611 TEMPORARY COLOSTOMY	4849 PULL-THRU RECT RESEC NEC
4612 TEMPORARY COLOSTOMY	485 ABD-PERINEAL RECT RESECT
4613 PERMANENT COLOSTOMY	4861 TRANSSAC RECTOSIGMOIDECT
4620 ILEOSTOMY NOS	4862 ANT RECT RESECT W COLOST
4621 TEMPORARY ILEOSTOMY	4863 ANTERIOR RECT RESECT NEC
4622 CONTINENT ILEOSTOMY	4864 POSTERIOR RECT RESECTION

4623 PERMANENT ILEOSTOMY NEC	4865 DUHAMEL RECTAL RESECTION
4866 DUHAMEL RECTAL RESECTION	5026 ABLTN LIVER LES/TISS NEC OCT06-
4869 RECTAL RESECTION NEC	5029 DESTRUC HEPATIC LES NEC
4871 SUTURE OF RECTAL LACER	503 HEPATIC LOBECTOMY
4872 CLOSURE OF PROCTOSTOMY	504 TOTAL HEPATECTOMY
4873 CLOSE RECTAL FIST NEC	5051 AUXILIARY LIVER TRANSPL
4874 RECTORECTOSTOMY	5059 LIVER TRANSPLANT NEC
4875 ABDOMINAL PROCTOPEXY	5061 CLOSURE LIVER LACERAT
4876 PROCTOPEXY NEC	5069 LIVER REPAIR NEC
4879 REPAIR OF RECTUM NEC	5102 TROCAR CHOLECYSTOSTOMY
4881 PERIRECTAL INCISION	5103 CHOLECYSTOSTOMY NEC
4882 PERIRECTAL EXCISION	5104 CHOLECYSTOTOMY NEC
4891 INCIS RECTAL STRICTURE	5113 OPEN BILIARY TRACT BX
4892 ANORECTAL MYOMECTOMY	5119 BILIARY TR DX PROC NEC
4893 REPAIR PERIRECT FISTULA	5121 OTH PART CHOLECYSTECTOMY
4899 RECTAL PERIRECT OP NEC	5122 CHOLECYSTECTOMY
4901 INCIS PERIANAL ABSCESS	5123 LAPAROSCOPIC CHOLECYSTEC
4902 PERIANAL INCISION NEC	5124 LAP PART CHOLECYSTECTOMY
4904 PERIANAL EXCISION NEC	5131 GB-TO-HEPAT DUCT ANAST
4911 ANAL FISTULOTOMY	5132 GB-TO-INTESTINE ANASTOM
4912 ANAL FISTULECTOMY	5133 GB-TO-PANCREAS ANASTOM
493 ANAL/PERIAN DX PROC NEC	5134 GB-TO-STOMACH ANASTOMOS
4939 OTHER DESTRUC ANUS LES	5135 GALLBLADDER ANASTOM NEC
4944 HEMORRHOID CRYOTHERAPY	5136 CHOLEDOCHOENTEROSTOMY
4945 HEMORRHOID LIGATION	5137 HEPATIC DUCT-GI ANASTOM
4946 HEMORRHOIDECTOMY	5139 BILE DUCT ANASTOMOS NEC
4949 HEMORRHOID PROCEDURE NEC	5141 CDE FOR CALCULUS REMOV
4951 LEFT LAT SPHINCTEROTOMY	5142 CDE FOR OBSTRUCTION NEC
4952 POST SPHINCTEROTOMY	5143 CHOLEDOCHOHEPAT INTUBAT
4959 ANAL SPHINCTEROTOMY NEC	5149 INCIS OBSTR BILE DUC NEC
496 EXCISION OF ANUS	5151 COMMON DUCT EXPLORATION
4971 SUTURE ANAL LACERATION	5159 BILE DUCT INCISION NEC
4972 ANAL CERCLAGE	5161 EXCIS CYST DUCT REMNANT
4973 CLOSURE OF ANAL FISTULA	5162 EXCIS AMPULLA OF VATER
4974 GRACILIS MUSC TRANSPLAN	5163 COMMON DUCT EXCIS NEC
4975 IMPL OR REV ART ANAL SPH	5169 BILE DUCT EXCISION NEC
4976 REMOV ART ANAL SPHINCTER	5171 SIMPLE SUT-COMMON DUCT
4979 ANAL SPHINCT REPAIR NEC	5172 CHOLEDOCHOPLASTY
4991 INCISION OF ANAL SEPTUM	5179 BILE DUCT REPAIR NEC
4992 INSERT SUBQ ANAL STIMUL	5181 SPHINCTER OF ODDI DILAT
4993 ANAL INCISION NEC	5182 PANCREAT SPHINCTEROTOM
4994 REDUCTION ANAL PROLAPSE	5183 PANCREAT SPHINCTEROPLAS
4995 CONTROL ANAL HEMORRHAGE	5189 SPHINCT OF ODDI OP NEC
4999 ANAL OPERATION NEC	5191 REPAIR GB LACERATION
500 HEPATOTOMY	5192 CLOSURE CHOLECYSTOSTOMY
5012 OPEN LIVER BIOPSY	5193 CLOS BILIARY FISTUL NEC
5019 HEPATIC DX PROC NEC	5194 REVIS BILE TRACT ANASTOM
5021 MARSUPIALIZAT LIVER LES	5195 REMOVE BILE DUCT PROSTH
5022 PARTIAL HEPATECTOMY	5199 BILIARY TRACT OP NEC
5023 OPN ABLTN LIVER LES/TISS OCT06-	5201 CATH DRAIN-PANCREAT CYST
5024 PERC ABLTN LIVER LES/TIS OCT06-	5209 PANCREATOTOMY NEC

5025 LAP ABLTN LIVER LES/TISS OCT06-	5212 OPEN PANCREATIC BIOPSY
5219 PANCREATIC DX PROC NEC	5412 REOPEN RECENT LAP SITE
522 PANCREATIC DX PROC NEC	5419 LAPAROTOMY NEC
5222 OTHER DESTRU PANCREA LES	5421 LAPAROSCOPY
523 PANCREAT CYST MARSUPIALI	5422 ABDOMINAL WALL BIOPSY
524 INT DRAIN PANCREAT CYST	5423 PERITONEAL BIOPSY
5251 PROXIMAL PANCREATECTOMY	5429 ABD REGION DX PROC NEC
5252 DISTAL PANCREATECTOMY	543 DESTRUCT ABD WALL LESION
5253 RAD SUBTOT PANCREATECTOM	544 DESTRUCT PERITONEAL TISS
5259 PARTIAL PANCREATECT NEC	545 DESTRUCT PERITONEAL TISS
526 TOTAL PANCREATECTOMY	5451 LAP PERITON ADHESIOLYSIS
527 RAD PANCREATICODUODENECT	5459 OTH PERITON ADHESIOLYSIS
5280 PANCREAT TRANSPLANT NOS	5461 RECLOSE POST OP DISRUPT
5281 REIMPLANT PANCREATIC TIS	5462 DELAYED CLOS ABD WOUND
5282 PANCREATIC HOMOTRANSPLAN	5463 ABD WALL SUTURE NEC
5283 PANCREATIC HETEROTRANSPL	5464 PERITONEAL SUTURE
5291 TRNSPLNT ISLETS LANG NOS	5471 REPAIR OF GASTROSCHISIS
5292 CANNULATION PANCREA DUC	5472 ABDOMEN WALL REPAIR NEC
5295 PANCREATIC REPAIR NEC	5473 PERITONEAL REPAIR NEC
5296 PANCREATIC ANASTOMOSIS	5474 OMENTAL REPAIR NEC
5299 PANCREATIC OPERATION NEC	5475 MESENTERIC REPAIR NEC
5300 UNILAT ING HERN REP NOS	5492 REMOVE FB FROM PERITON
5301 REPAIR DIRECT ING HERNIA	5493 CREATE CUTANPERITON FIST
5302 REPAIR INDIR ING HERNIA	5494 CREAT PERITONEOVAS SHUNT
5303 DIR ING HERNIA REP-GRAFT	5495 PERITONEAL INCISION
5304 IND ING HERNIA REP-GRAFT	5501 NEPHROTOMY
5305 ING HERNIA REP-GRAFT NOS	5502 NEPHROSTOMY
5310 BILAT ING HERNIA REP NOS	5503 PERCU NEPHROSTM W/O FRAG
5311 BILAT DIR ING HERN REP	5504 PERCU NEPHROSTMY W FRAG
5312 BILAT IND ING HERN REP	5511 PYELOTOMY
5313 BIL DIR/IND ING HRN REP	5512 PYELOSTOMY
5314 BIL DIR ING HRN REP-GRFT	5524 OPEN RENAL BIOPSY
5315 BIL IND ING HRN REP-GRFT	5529 RENAL DIAGNOST PROC NEC
5316 BIL DIR/IND ING HERN-PRO	5531 RENAL LES MARSUPIALIZAT
5317 BIL ING HRN REP-GRFT NOS	5532 OPN ABLTN RENAL LES/TISS OCT06-
5321 UNIL FEMOR HRN REP-GRFT	5533 PERC ABLTN RENL LES/TISS OCT06-
5329 UNIL FEMOR HERN REP NEC	5534 LAP ABLTN RENAL LES/TISS OCT06-
5331 BIL FEM HERN REPAIR-GRFT	5535 ABLTN RENAL LES/TISS NEC OCT06-
5339 BIL FEM HERN REPAIR NEC	5539 LOC DESTR RENAL LES NEC
5341 UMBIL HERNIA REPAIR-GRFT	554 PARTIAL NEPHRECTOMY
5349 UMBIL HERNIA REPAIR NEC	5551 NEPHROURETERECTOMY
5351 INCISIONAL HERNIA REPAIR	5552 SOLITARY KIDNEY NEPHRECT
5359 ABD WALL HERN REPAIR NEC	5553 REJECTED KIDNEY NEPHRECT
5361 INCIS HERNIA REPAIR-GRFT	5554 BILATERAL NEPHRECTOMY
5369 ABD HERN REPAIR-GRFT NEC	5561 RENAL AUTOTRANSPLANT
537 ABD REPAIR-DIAPHR HERNIA	5569 KIDNEY TRANSPLANT NEC
5380 THOR REP-DIAPH HERN NOS	557 NEPHROPEXY
5381 DIAPHRAGMATIC PLICATION	5581 SUTURE KIDNEY LACERATION
5382 PARASTERN HERNIA REPAIR	5582 CLOSE NEPHROST & PYELOST
539 OTHER HERNIA REPAIR	5583 CLOSE RENAL FISTULA NEC
540 ABDOMINAL WALL INCISION	5584 REDUCE RENAL PEDICL TORS

5411 EXPLORATORY LAPAROTOMY	5585 SYMPHYSIOTOMY
5586 RENAL ANASTOMOSIS	5779 TOTAL CYSTECTOMY NEC
5587 CORRECT URETEROPELV JUNC	5781 SUTURE BLADDER LACERAT
5589 RENAL REPAIR NEC	5782 CYSTOSTOMY CLOSURE
5591 RENAL DECAPSULATION	5783 ENTEROVESICO FIST REPAIR
5597 IMPLANT MECHANIC KIDNEY	5784 VESIC FISTULA REPAIR NEC
5598 REMOV MECHANICAL KIDNEY	5785 CYSTOURETHROPLASTY
5599 RENAL OPERATION NEC	5786 BLADDER EXSTROPHY REPAIR
560 TU REMOV URETER OBSTRUCT	5787 BLADDER RECONSTRUCTION
561 URETERAL MEATOTOMY	5788 BLADDER ANASTOMOSIS NEC
562 URETEROTOMY	5789 BLADDER REPAIR NEC
5634 OPEN URETERAL BIOPSY	5791 BLADDER SPHINCTEROTOMY
5639 URETERAL DX PROCEDUR NEC	5793 CONTROL BLADD HEMORRHAGE
5640 URETERECTOMY NOS	5796 IMPLANT BLADDER STIMULAT
5641 PARTIAL URETERECTOMY	5797 REPLACE BLADDER STIMULAT
5642 TOTAL URETERECTOMY	5798 REMOVE BLADDER STIMULAT
5651 FORM CUTAN ILEOURETEROST	5799 BLADDER OPERATION NEC
5652 REVIS CUTAN ILEOURETEROS	580 URETHROTOMY
5661 FORM CUTAN URETEROSTOMY	581 URETHRAL MEATOTOMY
5662 REVIS CUTAN URETEROS NEC	5841 SUTURE URETHRAL LACERAT
5671 URIN DIVERSION TO BOWEL	5842 URETHROSTOMY CLOSURE
5672 REVIS URETEROENTEROSTOMY	5843 CLOSE URETH FISTULA NEC
5673 NEPHROCYSTANASTOMOSI NOS	5844 URETHRAL REANASTOMOSIS
5674 URETERONEOCYSTOSTOMY	5845 HYPO-EPISPADIUS REPAIR
5675 TRANSURETEROURETEROSTOMY	5846 URETH RECONSTRUCTION NEC
5679 URETERAL ANASTOMOSIS NEC	5847 URETHRAL MEATOPLASTY
5681 INTRALUM URETE ADHESIOLY	5849 URETHRAL REPAIR NEC
5682 SUTURE URETERAL LACERAT	585 URETH STRICTURE RELEASE
5683 URETEROSTOMY CLOSURE	5891 PERIURETHRAL INCISION
5684 CLOSE URETER FISTULA NEC	5892 PERIURETHRAL EXCISION
5685 URETEROPEXY	5893 IMPLT ARTF URIN SPHINCT
5686 REMOVE URETERAL LIGATURE	5899 URETH/PERIURETH OP NEC
5689 REPAIR OF URETER NEC	5900 RETROPERIT DISSECT NOS
5692 IMPLANT URETERAL STIMUL	5901 RETROPERIT DISSECT NOS
5693 REPLACE URETERAL STIMUL	5902 PERIREN ADHESIOLYS NEC
5694 REMOVE URETERAL STIMULAT	5903 LAP LYS PERIREN/URET ADH
5695 LIGATION OF URETER	5909 PERIREN/URETER INCIS NEC
5699 URETERAL OPERATION NEC	5911 OTH LYS PERIVES ADHESIO
5712 CYSTOTOMY & ADHESIOLYSIS	5912 LAP LYS PERIVESURETH ADH
5718 OTHER SUPRAPU CYSTOSTOMY	5919 PERIVESICAL INCISION NEC
5719 CYSTOTOMY NEC	5921 PERIREN/URETERAL BIOPSY
5721 VESICOSTOMY	5929 PERIREN/URET DX PROC NEC
5722 REVISE CLO VESICOSTOMY	593 URETHROVES JUNCT PLICAT
5733 CLOS TRANSURETH BLADD BX	594 SUPRAPUBIC SLING OP
5734 OPEN BLADDER BIOPSY	595 RETROPUBIC URETH SUSPENS
5739 BLADDER DIAGNOS PROC NEC	596 PARAURETHRAL SUSPENSION
5741 TU ADHESIOLYSIS BLADDER	5971 LEVATOR MUSC SUSPENSION
5749 TU DESTRUC BLADD LES NEC	5979 URIN INCONTIN REPAIR NEC
5751 EXCISION OF URACHUS	5991 PERIREN/VESICLE EXCISION
5759 BLADDER LES DESTRUCT NEC	5992 PERIREN/VESICLE OP NEC
576 PARTIAL CYSTECTOMY	600 INCISION OF PROSTATE

5771 RADICAL CYSTECTOMY<br>6014 OPEN SEMINAL VESICLES BX<br>6015 PERIPROSTATIC BIOPSY<br>6018 PROSTATIC DX PROCED NEC<br>6019 SEMIN VES DX PROCED NEC<br>602 SEMIN VES DX PROCED NEC<br>6021 TRANSURETH PROSTATECTOMY<br>6029 OTH TRANSURETH PROSTATEC<br>603 SUPRAPUBIC PROSTATECTOMY<br>604 RETROPUBIC PROSTATECTOMY<br>605 RADICAL PROSTATECTOMY<br>6061 LOS EXCIS PROSTATIC LES<br>6062 PERINEAL PROSTATECTOMY<br>6069 PROSTATECTOMY NEC<br>6072 SEMINAL VESICLE INCISION<br>6073 SEMINAL VESICLE EXCISION<br>6079 SEMINAL VESICLE OP NEC<br>6081 PERIPROSTATIC INCISION<br>6082 PERIPROSTATIC EXCISION<br>6093 REPAIR OF PROSTATE<br>6094 CONTROL PROSTATE HEMORR<br>6095 TRANS BAL DIL PROS URETH<br>6096 TU DESTR PROSTATE BY MT<br>6097 OTH TU DESTR PROS - RT<br>6099 PROSTATIC OPERATION NEC<br>612 EXCISION OF HYDROCELE<br>6142 SCROTAL FISTULA REPAIR<br>6149 SCROTUM/TUNIC REPAIR NEC<br>6192 EXCISION TUNICA LES NEC<br>6199 SCROTUM & TUNICA OP NEC<br>620 INCISION OF TESTES<br>6212 OPEN TESTICULAR BIOPSY<br>6219 TESTES DX PROCEDURE NEC<br>622 TESTICULAR LES DESTRUCT<br>623 UNILATERAL ORCHIECTOMY<br>6241 REMOVE BOTH TESTES<br>6242 REMOVE SOLITARY TESTIS<br>625 ORCHIOPEXY<br>6261 SUTURE TESTICULAR LACER<br>6269 TESTICULAR REPAIR NEC<br>627 INSERT TESTICULAR PROSTH<br>6299 TESTICULAR OPERATION NEC<br>6309 SPERMAT CORD/VAS DX NEC<br>631 EXC SPERMATIC VARICOCELE<br>632 EXCISE EPIDIDYMIS CYST<br>633 EXCISE CORD/EPID LES NEC<br>634 EPIDIDYMECTOMY<br>6351 SUTURE CORD & EPID LACER<br>6353 TRANSPLANT SPERMAT CORD<br>6359 CORD & EPIDID REPAIR NEC<br>6381 SUTURE VAS & EPIDID LAC	6012 OPEN PROSTATIC BIOPSY<br>6383 EPIDIDYMOVASOSTOMY<br>6385 REMOV VAS DEFERENS VALVE<br>6389 VAS & EPIDIDY REPAIR NEC<br>6392 EPIDIDYMOTOMY<br>6393 SPERMATIC CORD INCISION<br>6394 SPERM CORD ADHESIOLYSIS<br>6395 INSERT VALVE IN VAS DEF<br>6399 CORD/EPID/VAS OPS NEC<br>640 CIRCUMCISION<br>6411 PENILE BIOPSY<br>642 LOCAL EXCIS PENILE LES<br>643 AMPUTATION OF PENIS<br>6441 SUTURE PENILE LACERATION<br>6442 RELEASE OF CHORDEE<br>6443 CONSTRUCTION OF PENIS<br>6444 RECONSTRUCTION OF PENIS<br>6445 REPLANTATION OF PENIS<br>6449 PENILE REPAIR NEC<br>645 SEX TRANSFORMAT OP NEC<br>6492 INCISION OF PENIS<br>6493 DIVISION OF PENILE ADHES<br>6495 INS NONINFL PENIS PROSTH<br>6496 REMOVE INT PENILE PROSTH<br>6497 INS INFLATE PENIS PROSTH<br>6498 PENILE OPERATION NEC<br>6499 MALE GENITAL OP NEC<br>650 MALE GENITAL OP NEC<br>6501 LAPAROSCOPIC OOPHOROTOMY<br>6509 OTHER OOPHOROTOMY<br>6511 OVARIAN ASPIRAT BIOPSY<br>6512 OVARIAN BIOPSY NEC<br>6513 LAP BIOPSY OF OVARY<br>6514 OTH LAP DX PROC OVARIES<br>6519 OVARIAN DX PROCEDURE NEC<br>6521 OVARIAN CYST MARSUPIALIZ<br>6522 OVARIAN WEDGE RESECTION<br>6523 LAP MARSUP OVARIAN CYST<br>6524 LAP WEDGE RESECT OVARY<br>6525 OTH LAP LOC EXC DEST OVA<br>6529 LOCAL DESTR OVA LES NEC<br>653 LOCAL DESTR OVA LES NEC<br>6531 LAP UNILAT OOPHORECTOMY<br>6539 OTH UNILAT OOPHORECTOMY<br>654 OTH UNILAT OOPHORECTOMY<br>6541 LAP UNI SALPINGO-OOPHOR<br>6549 OTH UNI SALPINGO-OOPHOR<br>6551 OTH REMOVE BOTH OVARIES<br>6552 OTH REMOVE REMAIN OVARY<br>6553 LAP REMOVE BOTH OVARIES<br>6554 LAP REMOVE REMAIN OVARY

6382 POSTOP VAS RECONSTRUCT<br>6562 OTH REMOVE REM OVA/TUBE<br>6563 LAP REMOVE OVARIES/TUBES<br>6564 LAP REMOVE REM OVA/TUBE<br>6571 OTH SIMPLE SUTURE OVARY<br>6572 OTH REIMPLANT OF OVARY<br>6573 OTH SALPINGO-OOPHOROPLAS<br>6574 LAP SIMPLE SUTURE OVARY<br>6575 LAP REIMPLANT OF OVARY<br>6576 LAP SALPINGO-OOPHOROPLAS<br>6579 REPAIR OF OVARY NEC<br>658 REPAIR OF OVARY NEC<br>6581 LAP ADHESIOLYS OVA/TUBE<br>6589 ADHESIOLYSIS OVARY/TUBE<br>6591 ASPIRATION OF OVARY<br>6592 TRANSPLANTATION OF OVARY<br>6593 MANUAL RUPT OVARIAN CYST<br>6594 OVARIAN DENERVATION<br>6595 OVARIAN TORSION RELEASE<br>6599 OVARIAN OPERATION NEC<br>660 OVARIAN OPERATION NEC<br>6601 SALPINGOTOMY<br>6602 SALPINGOSTOMY<br>6611 FALLOPIAN TUBE BIOPSY<br>6619 FALLOP TUBE DX PROC NEC<br>6621 BILAT ENDOSC CRUSH TUBE<br>6622 BILAT ENDOSC DIVIS TUBE<br>6629 BILAT ENDOS OCC TUBE NEC<br>6631 BILAT TUBAL CRUSHING NEC<br>6632 BILAT TUBAL DIVISION NEC<br>6639 BILAT TUBAL DESTRUCT NEC<br>664 TOTAL UNILAT SALPINGECT<br>6651 REMOVE BOTH FALLOP TUBES<br>6652 REMOVE SOLITARY FAL TUBE<br>6661 DESTROY FALLOP TUBE LES<br>6662 REMOV TUBE & ECTOP PREG<br>6663 BILAT PART SALPINGEC NOS<br>6669 PARTIAL SALPINGECTOM NEC<br>6671 SIMPL SUTURE FALLOP TUBE<br>6672 SALPINGO-OOPHOROSTOMY<br>6673 SALPINGO-SALPINGOSTOMY<br>6674 SALPINGO-UTEROSTOMY<br>6679 FALLOP TUBE REPAIR NEC<br>6692 UNILAT FALLOP TUBE DESTR<br>6693 IMPL FALLOP TUBE PROSTH<br>6694 REMOV FALLOP TUBE PROSTH<br>6695 BLOW THERAPEUT INTO TUBE<br>6696 FALLOPIAN TUBE DILATION<br>6697 BURY FIMBRIAE IN UTERUS<br>6699 FALLOPIAN TUBE OP NEC<br>6711 ENDOCERVICAL BIOPSY	6561 OTH REMOVE OVARIES/TUBES<br>6719 CERVICAL DX PROCEDUR NEC<br>672 CONIZATION OF CERVIX<br>6731 CERVICAL CYST MARSUPIAL<br>6732 CERVICAL LES CAUTERIZAT<br>6733 CERVICAL LES CRYOTHERAPY<br>6739 CERVICAL LES DESTRUC NEC<br>674 AMPUTATION OF CERVIX<br>675 AMPUTATION OF CERVIX<br>6751 TRANSAB CERCLAGE CERVIX<br>6759 OTH REP INT CERVICAL OS<br>6761 SUTURE CERVICAL LACERAT<br>6762 CERVICAL FISTULA REPAIR<br>6769 CERVICAL REPAIR NEC<br>680 HYSTEROTOMY<br>6813 OPEN UTERINE BIOPSY<br>6814 OPEN UTERINE LIGAMENT BX<br>6815 CLOS UTERINE LIGAMENT BX<br>6816 CLOSED UTERINE BIOPSY<br>6819 UTERUS/ADNEX DX PROC NEC<br>6821 ENDOMET SYNECHIAE DIVIS<br>6822 INCISION UTERINE SEPTUM<br>6823 ENDOMETRIAL ABLATION<br>6829 UTERINE LES DESTRUCT NEC<br>683 UTERINE LES DESTRUCT NEC<br>6831 LAP SCERVIC HYSTERECTOMY<br>6839 OTH SUBTOT ABD HYSTERECT10/03<br>684 TOTAL ABD HYSTERECTOMY<br>6841 LAP TOTAL ABDOMINAL HYST 10/06<br>6849 TOTAL ABD HYST NEC/NOS OCT06-<br>685 VAGINAL HYSTERECTOMY<br>6851 LAP AST VAG HYSTERECTOMY<br>6859 VAG HYSTERECTOMY NEC/NOS<br>686 RADICAL ABD HYSTERECTOMY<br>6861 LAP RADICAL ABD HYST 10/06<br>6869 RADICAL ABD HYST NEC/NOS 10/06<br>687 RADICAL VAG HYSTERECTOMY<br>6871 LAP RADICAL VAGINAL HYST 10/06<br>6879 RADICAL VAG HYST NEC/NOS 10/06<br>688 PELVIC EVISCERATION<br>689 HYSTERECTOMY NEC/NOS<br>6901 D & C FOR PREG TERMINAT<br>6902 D & C POST DELIVERY<br>6909 D & C NEC<br>6911 D & C NEC<br>6919 DESTRUC UTER SUPPORT NEC<br>6921 INTERPOSIT OP UTERIN LIG<br>6922 UTERINE SUSPENSION NEC<br>6923 VAG REPAIR INVERS UTERUS<br>6929 UTERUS/ADNEXA REPAIR NEC<br>693 PARACERV UTERINE DENERV

6712 CERVICAL BIOPSY NEC	6941 SUTURE UTERINE LACERAT
6942 CLOSURE UTERINE FISTULA	719 OTHER FEMALE GENITAL OPS
6949 UTERINE REPAIR NEC	7394 PUBIOTOMY TO ASSIST DEL
6951 ASPIRAT CURET-PREG TERMI	7399 OPS ASSISTING DELIV NEC
6952 ASPIRAT CURET-POST DELIV	740 CLASSICAL C-SECTION
6995 INCISION OF CERVIX	741 LOW CERVICAL C-SECTION
6997 REMOVE PENETRAT CERV FB	742 EXTRAPERITONEAL C-SECT
6998 UTERINE SUPPORT OP NEC	743 REM EXTRATUB ECTOP PREG
6999 UTERINE OPERATION NEC	744 CESAREAN SECTION NEC
7012 CULDOTOMY	7491 HYSTEROTOMY TO TERMIN PG
7013 INTRALUM VAG ADHESIOLYS	7499 CESAREAN SECTION NOS
7014 VAGINOTOMY NEC	7536 CORRECTION FETAL DEFECT
7023 CUL-DE-SAC BIOPSY	7550 REPAIR OB LAC UTERUS NOS
7024 VAGINAL BIOPSY	7551 REPAIR OB LACERAT CERVIX
7029 VAGIN/CUL-DE-SAC DX NEC	7552 REPAIR OB LAC CORP UTERI
7031 HYMENECTOMY	7561 REPAIR OB LAC BLAD/URETH
7032 EXCIS CUL-DE-SAC LESION	7593 SURG CORR INVERT UTERUS
7033 EXCISION VAGINAL LESION	7599 OBSTETRIC OPERATION NEC
704 VAGINAL OBLITERATION	7601 FACIAL BONE SEQUESTRECT
7050 CYSTOCEL/RECTOCEL REPAIR	7609 FACIAL BONE INCISION NEC
7051 CYSTOCELE REPAIR	7611 FACIAL BONE BIOPSY
7052 RECTOCELE REPAIR	7619 FACIAL BONE DX PROC NEC
7061 VAGINAL CONSTRUCTION	762 DESTRUCT FACIAL BONE LES
7062 VAGINAL RECONSTRUCTION	7631 PARTIAL MANDIBULECTOMY
7071 SUTURE VAGINA LACERATION	7639 PART FACIAL OSTECTOM NEC
7072 REPAIR COLOVAGIN FISTULA	7641 TOT MANDIBULEC W RECONST
7073 REPAIR RECTOVAG FISTULA	7642 TOTAL MANDIBULECTOMY NEC
7074 REP VAGINOENT FISTUL NEC	7643 MANDIBULAR RECONST NEC
7075 REPAIR VAG FISTULA NEC	7644 TOT FACE OSTECT W RECONS
7076 HYMENORRHAPHY	7645 TOT FACE BONE OSTECT NEC
7077 VAGINAL SUSPENS & FIXAT	7646 FACIAL BONE RECONSTR NEC
7079 VAGINAL REPAIR NEC	765 TEMPOROMAND ARTHROPLASTY
708 VAGINAL VAULT OBLITERAT	7661 CL OSTEOPLASTY MAND RAMI
7091 VAGINAL OPERATION NEC	7662 OPEN OSTEOPLAS MAND RAMI
7092 CUL-DE-SAC OPERATION NEC	7663 OSTEOPLASTY MANDIBLE BDY
7101 VULVAR ADHESIOLYSIS	7664 MAND ORTHOGNATHIC OP NEC
7109 INCIS VULVA/PERINEUM NEC	7665 SEG OSTEOPLASTY MAXILLA
7111 VULVAR BIOPSY	7666 TOT OSTEOPLASTY MAXILLA
7119 VULVAR DIAGNOS PROC NEC	7667 REDUCTION GENIOPLASTY
7122 INCISE BARTHOLIN"S GLAND	7668 AUGMENTATION GENIOPLASTY
7123 BARTHOLIN GLAND MARSUP	7669 FACIAL BONE REPAIR NEC
7124 DESTRUC BARTHOLIN GLAND	7670 REDUCTION FACIAL FX NOS
7129 BARTHOLIN"S GLAND OP NEC	7672 OPN REDUCT MALAR/ZYGO FX
713 LOCAL VULVAR EXCIS NEC	7674 OPEN REDUCT MAXILLARY FX
714 OPERATIONS ON CLITORIS	7676 OPEN REDUCT MANDIBLE FX
715 RADICAL VULVECTOMY	7677 OPEN REDUCT ALVEOLAR FX
7161 UNILATERAL VULVECTOMY	7679 OPEN REDUCT FACE FX NEC
7162 BILATERAL VULVECTOMY	7691 BONE GRAFT TO FACE BONE
7171 SUTURE VULVAR LACERATION	7692 SYN IMPLANT TO FACE BONE
7172 REPAIR VULVAR FISTULA	7694 OPEN REDUCT TM DISLOCAT
7179 VULVAR/PERIN REPAIR NEC	7697 REMOVE INT FIX FACE BONE

718 OTHER VULVAR OPERATIONS	7699 FACIAL BONE/JNT OP NEC
7700 SEQUESTRECTOMY NOS	7752 BUNIONECT/SFT/ARTHRODES
7701 CHEST CAGE SEQUESTREC	7753 OTH BUNIONECT W SFT CORR
7702 HUMERUS SEQUESTRECTOMY	7754 EXC CORRECT BUNIONETTE
7703 RADIUS & ULNA SEQUESTREC	7756 REPAIR OF HAMMER TOE
7704 METACARP/CARP SEQUESTREC	7757 REPAIR OF CLAW TOE
7705 FEMORAL SEQUESTRECTOMY	7758 OTH EXC, FUS, REPAIR TOE
7706 PATELLAR SEQUESTRECTOMY	7759 BUNIONECTOMY NEC
7707 TIBIA/FIBULA SEQUESTREC	7760 LOC EXC BONE LESION NOS
7708 METATAR/TAR SEQUESTREC	7761 EXC CHEST CAGE BONE LES
7709 SEQUESTRECTOMY NEC	7762 LOC EXC BONE LES HUMERUS
7710 OTHER BONE INCISION NOS	7763 LOC EXC LES RADIUS/ULNA
7711 OTHER CHEST CAGE INCIS	7764 LOC EXC LES METACAR/CAR
7712 OTHER HUMERUS INCISION	7765 LOC EXC BONE LES FEMUR
7713 OTHER RADIUS/ULNA INCIS	7766 LOC EXC BONE LES PATELLA
7714 OTH METACARP/CARP INCIS	7767 LOC EXC LES TIBIA/FIBULA
7715 OTHER FEMORAL INCISION	7768 LOC EXC LES METATAR/TAR
7716 OTHER PATELLAR INCISION	7769 LOC EXC BONE LESION NEC
7717 OTHER TIBIA/FIBULA INCIS	7770 EXCISE BONE FOR GRFT NOS
7718 OTH METATARS/TARS INCIS	7771 EX CHEST CAGE BONE-GFT
7719 BONE INCIS W/O DIV NEC	7772 EXCISE HUMERUS FOR GRAFT
7720 WEDGE OSTEOTOMY NOS	7773 EXCIS RADIUS/ULNA-GRAFT
7721 CHEST CAGE WEDG OSTEOTOM	7774 EXCIS METACAR/CAR-GRAFT
7722 HUMERUS WEDGE OSTEOTOMY	7775 EXCISE FEMUR FOR GRAFT
7723 RADIUS/ULNA WEDG OSTEOTO	7776 EXCISE PATELLA FOR GRAFT
7724 METACAR/CAR WEDG OSTEOTO	7777 EXCISE TIB/FIB FOR GRAFT
7725 FEMORAL WEDGE OSTEOTOMY	7778 EXCIS METATAR/TAR-GRAFT
7726 PATELLAR WEDGE OSTEOTOMY	7779 EXCISE BONE FOR GFT NEC
7727 TIBIA/FIBUL WEDG OSTEOT	7780 OTH PART OSTECTOMY NOS
7728 METATAR/TAR WEDG OSTEOT	7781 OTH CHEST CAGE OSTECTOMY
7729 WEDGE OSTEOTOMY NEC	7782 PARTIAL HUMERECTOMY NEC
7730 OTHER BONE DIVISION NOS	7783 PART OSTECT-RADIUS/ULNA
7731 CHEST CAGE BONE DIV NEC	7784 PART OSTECT-METACAR/CAR
7732 HUMERUS DIVISION NEC	7785 PART OSTECTOMY-FEMUR
7733 RADIUS/ULNA DIVISION NEC	7786 PARTIAL PATELLECTOMY
7734 METACAR/CAR DIVISION NEC	7787 PART OSTECT-TIBIA/FIBULA
7735 FEMORAL DIVISION NEC	7788 PART OSTECT-METATAR/TAR
7736 PATELLAR DIVISION NEC	7789 PARTIAL OSTECTOMY NEC
7737 TIBIA/FIBULA DIV NEC	7790 TOTAL OSTECTOMY NOS
7738 METATAR/TAR DIVISION NEC	7791 TOT CHEST CAGE OSTECTOMY
7739 BONE DIVISION NEC	7792 TOTAL OSTECTOMY-HUMERUS
7740 BONE BIOPSY NOS	7793 TOT OSTECT-RADIUS/ULNA
7741 CHEST CAGE BONE BIOPSY	7794 TOT OSTECT-METACARP/CARP
7742 HUMERUS BIOPSY	7795 TOT OSTECTOMY-FEMUR
7743 RADIUS & ULNA BIOPSY	7796 TOTAL PATELLECTOMY
7744 METACARPAL/CARPAL BIOPSY	7797 TOT OSTECT-TIBIA/FIBULA
7745 FEMORAL BIOPSY	7798 TOT OSTECT-METATARS/TARS
7746 PATELLAR BIOPSY	7799 TOTAL OSTECTOMY NEC
7747 TIBIA & FIBULA BIOPSY	7800 BONE GRAFT NOS
7748 METATARSAL/TARSAL BIOPSY	7801 BONE GRAFT TO CHEST CAGE
7749 BONE BIOPSY NEC	7802 BONE GRAFT TO HUMERUS

7751 BUNIONECT/SFT/OSTEOTOMY	7803 BONE GRAFT-RADIUS/ULNA
7804 BONE GRFT TO METACAR/CAR	7858 INT FIXATION-METATAR/TAR
7805 BONE GRAFT TO FEMUR	7859 INT FIX-NO FX REDUCT NEC
7806 BONE GRAFT TO PATELLA	7860 REMOVE IMP DEVICE NOS
7807 BONE GRAFT-TIBIA/FIBULA	7861 REMOV IMP DEV-CHEST CAGE
7808 BONE GRAFT-METATAR/TAR	7862 REMOVE IMPL DEV-HUMERUS
7809 BONE GRAFT NEC	7863 REMOV IMP DEV-RADIUS/ULN
7810 APPLIC EXT FIX DEV NOS	7864 REMOV IMP DEV-METAC/CARP
7811 APPL EXT FIX-CHEST CAGE	7865 REMOVE IMP DEVICE-FEMUR
7812 APPLIC EXT FIX-HUMERUS	7866 REMOV IMP DEVICE-PATELLA
7813 APPL EXT FIX-RADIUS/ULNA	7867 REMOV IMP DEV-TIB/FIBULA
7814 APPL EXT FIX-METACAR/CAR	7868 REMOVE IMP DEV-METAT/TAR
7815 APPLIC EXT FIX DEV-FEMUR	7869 REMOVE IMPL DEVICE NEC
7816 APPL EXT FIX DEV-PATELLA	7870 OSTEOCLASIS NOS
7817 APPL EXT FIX-TIB/FIBULA	7871 OSTEOCLASIS-CHEST CAGE
7818 APPL EXT FIX-METATAR/TAR	7872 OSTEOCLASIS-HUMERUS
7819 APPLIC EXT FIX DEV NEC	7873 OSTEOCLASIS-RADIUS/ULNA
7820 LIMB SHORTEN PROC NOS	7874 OSTEOCLASIS-METACAR/CAR
7822 LIMB SHORT PROC-HUMERUS	7875 OSTEOCLASIS-FEMUR
7823 LIMB SHORTEN-RADIUS/ULNA	7876 OSTEOCLASIS-PATELLA
7824 LIMB SHORTEN-METACAR/CAR	7877 OSTEOCLASIS-TIBIA/FIBULA
7825 LIMB SHORT PROC-FEMUR	7878 OSTEOCLASIS-METATAR/TAR
7827 LIMB SHORTEN-TIB/FIBULA	7879 OSTEOCLASIS NEC
7828 LIMB SHORTEN-METATAR/TAR	7880 OTHER BONE DX PROC NOS
7829 LIMB SHORTEN PROC NEC	7881 OTH DX PROCED-CHEST CAGE
7830 LIMB LENGTHEN PROC NOS	7882 OTH DX PROCED-HUMERUS
7831 LIMB LENGTHEN PROC NOS	7883 OTH DX PROC-RADIUS/ULNA
7832 LIMB LENGTH PROC-HUMERUS	7884 OTH DX PROC-METACAR/CAR
7833 LIMB LENGTH-RADIUS/ULNA	7885 OTH DX PROCED-FEMUR
7834 LIMB LENGTH-METACAR/CAR	7886 OTH DX PROCED-PATELLA
7835 LIMB LENGTH PROC-FEMUR	7887 OTH DX PROC-TIBIA/FIBULA
7837 LIMB LENGTHEN-TIB/FIBULA	7888 OTH DX PROC-METATAR/TAR
7838 LIMB LENGTHN-METATAR/TAR	7889 OTHER BONE DX PROC NEC
7839 LIMB LENGTHEN PROC NEC	7890 INSERT BONE STIMUL NOS
7840 OTH BONE REPAIR/PLAST OP	7891 INSERT BONE STIMUL-CHEST
7841 OTH CHEST CAGE REP/PLAST	7892 INSERT BONE STIM-HUMERUS
7842 OTH HUMERUS REPAIR/PLAST	7893 INSER BONE STIM-RAD/ULNA
7843 OTH RAD/ULN REPAIR/PLAST	7894 INSER BONE STIM-META/CAR
7844 OTH METAC/CARP REP/PLAST	7895 INSERT BONE STIM-FEMUR
7845 OTH FEMUR REPAIR/PLASTIC	7896 INSERT BONE STIM-PATELLA
7846 OTH PATELLA REPAIR/PLAST	7897 INSER BONE STIM-TIB/FIB
7847 OTH TIB/FIB REPAIR/PLAST	7898 INSER BONE STIM-META/TAR
7848 OTH META/TAR REPA/PLAST	7899 INSERT BONE STIMUL NEC
7849 OTH BONE REPA/PLAST NEC	7910 CL FX REDUC-INT FIX NOS
7850 INT FIX W/O FX REDUC NOS	7911 CLOS RED-INT FIX HUMERUS
7851 INT FIXATION-CHEST CAGE	7912 CL RED-INT FIX RAD/ULNA
7852 INT FIXATION-HUMERUS	7913 CL RED-INT FIX METAC/CAR
7853 INT FIXATION-RADIUS/ULNA	7914 CLOSE RED-INT FIX FINGER
7854 INT FIXATION-METACAR/CAR	7915 CLOSED RED-INT FIX FEMUR
7855 INTERNAL FIXATION-FEMUR	7916 CL RED-INT FIX TIB/FIBU
7856 INTERNAL FIX-PATELLA	7917 CL RED-INT FIX METAT/TAR

7857 INT FIXATION-TIBIA/FIBUL	7918 CLOSE RED-INT FIX TOE FX
7919 CL FX REDUC-INT FIX NEC	7988 OPN REDUC DISLOC-FT/TOE
7920 OPEN FX REDUCTION NOS	7989 OPEN REDUC-DISLOCAT NEC
7921 OPEN REDUC-HUMERUS FX	7990 UNSPEC OP BONE INJ NOS
7922 OPEN REDUC-RADIUS/ULN FX	7991 HUMERUS INJURY OP NOS
7923 OPEN REDUC-METAC/CAR FX	7992 RADIUS/ULNA INJ OP NOS
7924 OPEN REDUCTION-FINGER FX	7993 METACARP/CARP INJ OP NOS
7925 OPEN REDUCTION-FEMUR FX	7994 FINGER INJURY OP NOS
7926 OPEN REDUC-TIBIA/FIB FX	7995 FEMUR INJURY OP NOS
7927 OPEN REDUC-METAT/TARS FX	7996 TIBIA/FIBULA INJ OP NOS
7928 OPEN REDUCTION-TOE FX	7997 METATARS/TARS INJ OP NOS
7929 OPEN FX REDUCTION NEC	7998 TOE INJURY OPERATION NOS
7930 OPN FX RED W INT FIX NOS	7999 UNSPEC OP-BONE INJ NEC
7931 OPEN RED-INT FIX HUMERUS	8000 ARTHROT & PROS REMOV NOS
7932 OP RED-INT FIX RAD/ULNA	8001 ARTHROT/PROS REMOV-SHLDR
7933 OP RED-INT FIX METAC/CAR	8002 ARTHROT/PROS REMOV-ELBOW
7934 OPEN RED-INT FIX FINGER	8003 ARTHROT/PROS REMOV-WRIST
7935 OPEN REDUC-INT FIX FEMUR	8004 ARTHROT/PROS REMOV-HAND
7936 OP RED-INT FIX TIB/FIBUL	8005 ARTHROT/PROS REMOV-HIP
7937 OP RED-INT FIX METAT/TAR	8006 ARTHROT/PROS REMOV-KNEE
7938 OPEN REDUCT-INT FIX TOE	8007 ARTHROT/PROS REMOV-ANKLE
7939 OPN FX RED W INT FIX NEC	8008 ARTHROT/PROS REMOV-FOOT
7940 CLS REDUC-SEP EPIPHY NOS	8009 ARTHROT & PROS REMOV NEC
7941 CLOSE RED-HUMERUS EPIPHY	8010 OTHER ARTHROTOMY NOS
7942 CLS RED-RADIUS/UL EPIPHY	8011 OTH ARTHROTOMY-SHOULDER
7945 CLOSE REDUC-FEMUR EPIPHY	8012 OTH ARTHROTOMY-ELBOW
7946 CLS RED-TIBIA/FIB EPIPHY	8013 OTH ARTHROTOMY-WRIST
7949 CLS REDUC-SEP EPIPHY NEC	8014 OTH ARTHROTOMY-HAND/FNGR
7950 OPEN RED-SEP EPIPHY NOS	8015 OTH ARTHROTOMY-HIP
7951 OPN RED-SEP EPIPHY-HUMER	8016 OTH ARTHROTOMY-KNEE
7952 OP RED-RADIUS/ULN EPIPHY	8017 OTH ARTHROTOMY-ANKLE
7955 OPN RED-SEP EPIPHY-FEMUR	8018 OTH ARTHROTOMY-FOOT/TOE
7956 OP RED-TIBIA/FIB EPIPHYS	8019 OTHER ARTHROTOMY NEC
7959 OPEN RED-SEP EPIPHY NEC	8020 ARTHROSCOPY NOS
7960 OPEN FX SITE DEBRIDE NOS	8021 SHOULDER ARTHROSCOPY
7961 DEBRID OPEN FX-HUMERUS	8022 ELBOW ARTHROSCOPY
7962 DEBRID OPN FX-RADIUS/ULN	8023 WRIST ARTHROSCOPY
7963 DEBRID OPN FX-METAC/CAR	8024 HAND & FINGER ARTHROSCOP
7964 DEBRID OPN FX-FINGER	8025 HIP ARTHROSCOPY
7965 DEBRID OPN FX-FEMUR	8026 KNEE ARTHROSCOPY
7966 DEBRID OPN FX-TIBIA/FIB	8027 ANKLE ARTHROSCOPY
7967 DEBRID OPN FX-METAT/TAR	8028 FOOT & TOE ARTHROSCOPY
7968 DEBRID OPN FX-TOE	8029 ARTHROSCOPY NEC
7969 OPEN FX SITE DEBRIDE NEC	8040 JT STRUCTUR DIVISION NOS
7980 OPEN REDUC-DISLOCAT NOS	8041 SHOULDER STRUCT DIVISION
7981 OPN REDUC DISLOC-SHOULDR	8042 ELBOW STRUCTURE DIVISION
7982 OPEN REDUC-ELBOW DISLOC	8043 WRIST STRUCTURE DIVISION
7983 OPEN REDUC-WRIST DISLOC	8044 HAND JOINT STRUCT DIVIS
7984 OPN REDUC DISLOC-HAND	8045 HIP STRUCTURE DIVISION
7985 OPEN REDUC-HIP DISLOCAT	8046 KNEE STRUCTURE DIVISION
7986 OPEN REDUC-KNEE DISLOCAT	8047 ANKLE STRUCTURE DIVISION

7987 OPEN REDUC-ANKLE DISLOC	8048 FOOT JOINT STRUCT DIVIS
8049 JT STRUCTUR DIVISION NEC	8116 METATARSOPHALANGEAL FUS
805 JT STRUCTUR DIVISION NEC	8117 OTHER FUSION OF FOOT
8050 EXC/DEST INTVRT DISC NOS	8118 OTHER FUSION OF FOOT
8051 EXCISION INTERVERT DISC	8120 ARTHRODESIS NOS
8059 OTH EXC/DEST INTVRT DISC	8121 ARTHRODESIS OF HIP
806 EXCIS KNEE SEMILUN CARTL	8122 ARTHRODESIS OF KNEE
8070 SYNOVECTOMY-SITE NOS	8123 ARTHRODESIS OF SHOULDER
8071 SHOULDER SYNOVECTOMY	8124 ARTHRODESIS OF ELBOW
8072 ELBOW SYNOVECTOMY	8125 CARPORADIAL FUSION
8073 WRIST SYNOVECTOMY	8126 METACARPOCARPAL FUSION
8074 HAND SYNOVECTOMY	8127 METACARPOPHALANGEAL FUS
8075 HIP SYNOVECTOMY	8128 INTERPHALANGEAL FUSION
8076 KNEE SYNOVECTOMY	8129 ARTHRODESIS NEC
8077 ANKLE SYNOVECTOMY	8130 SPINAL REFUSION NOS
8078 FOOT SYNOVECTOMY	8131 REFUSION OF ATLAS-AXIS
8079 SYNOVECTOMY-SITE NEC	8132 REFUSION OF OTH CERV ANT
8080 DESTRUCT JOINT LES NOS	8133 REFUS OF OTH CERV POST
8081 DESTRUC-SHOULDER LES NEC	8134 REFUSION OF DORSAL ANT
8082 DESTRUC-ELBOW LESION NEC	8135 REFUSION OF DORSAL POST
8083 DESTRUC-WRIST LESION NEC	8136 REFUSION OF LUMBAR ANT
8084 DESTRUC-HAND JT LES NEC	8137 REFUSION OF LUMBAR LAT
8085 DESTRUCT-HIP LESION NEC	8138 REFUSION OF LUMBAR POST
8086 DESTRUCT-KNEE LESION NEC	8139 REFUSION OF SPINE NEC
8087 DESTRUC-ANKLE LESION NEC	8140 REPAIR OF HIP, NEC
8088 DESTRUC-FOOT JT LES NEC	8141 REPAIR OF HIP, NEC
8089 DESTRUCT JOINT LES NEC	8142 FIVE-IN-ONE KNEE REPAIR
8090 EXCISION OF JOINT NOS	8143 TRIAD KNEE REPAIR
8091 EXCISION OF SHOULDER NEC	8144 PATELLAR STABILIZATION
8092 EXCISION OF ELBOW NEC	8145 CRUCIATE LIG REPAIR NEC
8093 EXCISION OF WRIST NEC	8146 COLLATERL LIG REPAIR NEC
8094 EXCISION HAND JOINT NEC	8147 OTHER REPAIR OF KNEE
8095 EXCISION OF HIP NEC	8148 OTHER REPAIR OF KNEE
8096 EXCISION OF KNEE NEC	8149 OTHER REPAIR OF ANKLE
8097 EXCISION OF ANKLE NEC	8151 TOTAL HIP REPLACEMENT
8098 EXCISION FOOT JOINT NEC	8152 PARTIAL HIP REPLACEMENT
8099 EXCISION OF JOINT NEC	8153 REVISE HIP REPLACEMENT
8100 SPINAL FUSION NOS	8154 TOTAL KNEE REPLACEMENT
8101 ATLAS-AXIS FUSION	8155 REVISE KNEE REPLACEMENT
8102 OTHER CERVICAL FUS ANT	8156 TOTAL ANKLE REPLACEMENT
8103 OTHER CERVICAL FUS POST	8157 REPL JOINT OF FOOT, TOE
8104 DORSAL/DORSOLUM FUS ANT	8159 REV JT REPL LOW EXT NEC
8105 DORSAL/DORSOLUM FUS POST	8161 360 SPINAL FUSION
8106 LUMBAR/LUMBOSAC FUS ANT	8162 FUS/REFUS 2-3 VERTEBRAE
8107 LUMBAR/LUMBOSAC FUS LAT	8163 FUS/REFUS 4-8 VERTEBRAE
8108 LUMBAR/LUMBOSAC FUS POST	8164 FUS/REFUS 9 VERTEBRAE
8109 LUMBAR/LUMBOSAC FUS POST	8165 VERTEBROPLASTY (OCT 04)
8111 ANKLE FUSION	8166 KYPHOPLASTY (OCT 04)
8112 TRIPLE ARTHRODESIS	8169 OTH HIP REPAIR JAN80--SEP89 `0/05
8113 SUBTALAR FUSION	8171 ARTHROPLAS METACARP WIT
8114 MIDTARSAL FUSION	8172 ARTHROPLASTY METACAR W/O

8115 TARSOMETATARSAL FUSION	8173 TOTAL WRIST REPLACEMENT
8174 ARTHROPLASTY CARPAL WIT	8269 THUMB RECONSTRUCTION NEC
8175 ARTHROPLASTY CARPAL W/O	8271 HAND TEND PULLEY RECONST
8179 OTH REPAIR HAN/FIN/WRIS	8272 PLAST OP HND-MUS/FAS GRF
8180 TOTAL SHOULDER REPLACE	8279 PLAST OP HAND W GRFT NEC
8181 PARTIAL SHOULDER REPLACE	8281 TRANSFER OF FINGER
8182 REP RECUR SHLDER DISLOC	8282 REPAIR OF CLEFT HAND
8183 SHOULDER ARTHROPLAST NEC	8283 REPAIR OF MACRODACTYLY
8184 TOTAL ELBOW REPLACEMENT	8284 REPAIR OF MALLET FINGER
8185 ELBOW ARTHROPLASTY NEC	8285 OTHER TENODESIS OF HAND
8186 ELBOW ARTHROPLASTY NEC	8286 OTHER TENOPLASTY OF HAND
8187 ELBOW ARTHROPLASTY NEC	8289 HAND PLASTIC OP NEC
8193 SUTUR CAPSUL/LIGAMEN ARM	8291 LYSIS OF HAND ADHESIONS
8194 SUTURE CAPSUL/LIG ANK/FT	8299 HAND MUS/TEN/FAS/OPS NEC
8195 SUTUR CAPSUL/LIG LEG NEC	8301 TENDON SHEATH EXPLORAT
8196 OTHER REPAIR OF JOINT	8302 MYOTOMY
8197 REV JT REPL UPPER EXTREM	8303 BURSOTOMY
8198 OTHER JOINT DX PROCEDURE	8309 SOFT TISSUE INCISION NEC
8199 JOINT STRUCTURE OP NEC	8311 ACHILLOTENOTOMY
8201 EXPLOR TEND SHEATH-HAND	8312 ADDUCTOR TENOTOMY OF HIP
8202 MYOTOMY OF HAND	8313 OTHER TENOTOMY
8203 BURSOTOMY OF HAND	8314 FASCIOTOMY
8209 INC SOFT TISSUE HAND NEC	8319 SOFT TISSUE DIVISION NEC
8211 TENOTOMY OF HAND	8321 SOFT TISSUE BIOPSY
8212 FASCIOTOMY OF HAND	8329 SOFT TISSUE DX PROC NEC
8219 DIV SOFT TISSUE HAND NEC	8331 EXCIS LES TENDON SHEATH
8221 EXC LES TEND SHEATH HAND	8332 EXCIS LESION OF MUSCLE
8222 EXCISION HAND MUSCLE LES	8339 EXC LES SOFT TISSUE NEC
8229 EXC LES SFT TISS HND NEC	8341 TENDON EXCISION FOR GRFT
8231 BURSECTOMY OF HAND	8342 OTHER TENONECTOMY
8232 EXCIS HAND TEND FOR GRFT	8343 MUSC/FASC EXCIS FOR GRFT
8233 HAND TENONECTOMY NEC	8344 OTHER FASCIECTOMY
8234 EXC HND MUS/FAS FOR GRFT	8345 OTHER MYECTOMY
8235 HAND FASCIECTOMY NEC	8349 OTHER SOFT TISSUE EXCIS
8236 OTHER MYECTOMY OF HAND	835 BURSECTOMY
8239 HAND SOFT TISSUE EXC NEC	8361 TENDON SHEATH SUTURE
8241 SUTURE TENDN SHEATH HAND	8362 DELAYED TENDON SUTURE
8242 DELAY SUT FLEX TEND HAND	8363 ROTATOR CUFF REPAIR
8243 DELAY SUT HAND TEND NEC	8364 OTHER SUTURE OF TENDON
8244 SUTUR FLEX TEND HAND NEC	8365 OTHER MUSCLE/FASC SUTURE
8245 SUTURE HAND TENDON NEC	8371 TENDON ADVANCEMENT
8246 SUTURE HAND MUSCLE/FASC	8372 TENDON RECESSION
8251 HAND TENDON ADVANCEMENT	8373 TENDON REATTACHMENT
8252 HAND TENDON RECESSION	8374 MUSCLE REATTACHMENT
8253 HAND TENDON REATTACHMENT	8375 TENDON TRNSFR/TRANSPLANT
8254 HAND MUSCLE REATTACHMENT	8376 OTHER TENDON TRANSPOSIT
8255 CHNG HND MUS/TEN LNG NEC	8377 MUSCLE TRNSFR/TRANSPLANT
8256 TRANSPLANT HAND TEND NEC	8379 OTHER MUSCLE TRANSPOSIT
8257 TRANSPOSIT HAND TEND NEC	8381 TENDON GRAFT
8258 TRANSPLANT HAND MUSC NEC	8382 MUSCLE OR FASCIA GRAFT
8259 TRANSPOSIT HAND MUSC NEC	8383 TENDON PULLEY RECONSTRUC

8261 POLLICIZATION OPERATION	8384 CLUBFOOT RELEASE NEC
8385 MUSC/TEND LNG CHANGE NEC	8467 REVISE DISC PROST THORA 10/04
8386 QUADRICEPSPLASTY	8468 REVISE DISC PROSTH LUMB 10/04
8387 OTHER PLASTIC OPS MUSCLE	8469 REVISE DISC PROSTH NOS (OCT 04)
8388 OTHER PLASTIC OPS TENDON	8472 APP EXT FIX DEV-RING SYS OCT05-
8389 OTHER PLASTIC OPS FASCIA	8473 APP HYBRID EXT FIX DEV OCT05-
8391 ADHESIOLYSIS MUS/TEN/FAS	8491 AMPUTATION NOS
8392 INSERT SKEL MUSC STIMULA	8492 SEPARAT EQUAL JOIN TWIN
8393 REMOV SKEL MUSC STIMULAT	8493 SEPARAT UNEQUL JOIN TWIN
8399 MUS/TEN/FAS/BUR OP NEC	8499 MUSCULOSKELETAL OP NEC
8400 UPPER LIMB AMPUTAT NOS	8512 OPEN BREAST BIOPSY
8401 FINGER AMPUTATION	8520 BREAST TISSU DESTRUC NOS
8402 THUMB AMPUTATION	8521 LOCAL EXCIS BREAST LES
8403 AMPUTATION THROUGH HAND	8522 QUADRANT RESECT BREAST
8404 DISARTICULATION OF WRIST	8523 SUBTOTAL MASTECTOMY
8405 AMPUTATION THRU FOREARM	8524 EXC ECTOPIC BREAST TISSU
8406 DISARTICULATION OF ELBOW	8525 EXCISION OF NIPPLE
8407 AMPUTATION THRU HUMERUS	8531 UNILAT REDUCT MAMMOPLAST
8408 SHOULDER DISARTICULATION	8532 BILAT REDUCT MAMMOPLASTY
8409 FOREQUARTER AMPUTATION	8533 UNIL SUBQ MAMMECT-IMPLNT
8410 LOWER LIMB AMPUTAT NOS	8534 UNILAT SUBQ MAMMECT NEC
8411 TOE AMPUTATION	8535 BIL SUBQ MAMMECT-IMPLANT
8412 AMPUTATION THROUGH FOOT	8536 BILAT SUBQ MAMMECTOM NEC
8413 DISARTICULATION OF ANKLE	8541 UNILAT SIMPLE MASTECTOMY
8414 AMPUTAT THROUGH MALLEOLI	8542 BILAT SIMPLE MASTECTOMY
8415 BELOW KNEE AMPUTAT NEC	8543 UNILAT EXTEN SIMP MASTEC
8416 DISARTICULATION OF KNEE	8544 BILAT EXTEND SIMP MASTEC
8417 ABOVE KNEE AMPUTATION	8545 UNILAT RADICAL MASTECTOM
8418 DISARTICULATION OF HIP	8546 BILAT RADICAL MASTECTOMY
8419 HINDQUARTER AMPUTATION	8547 UNIL EXT RAD MASTECTOMY
8421 THUMB REATTACHMENT	8548 BIL EXTEN RAD MASTECTOMY
8422 FINGER REATTACHMENT	8550 AUGMENT MAMMOPLASTY NOS
8423 FOREARM/WRIST/HAND REATT	8553 UNILAT BREAST IMPLANT
8424 UPPER ARM REATTACHMENT	8554 BILATERAL BREAST IMPLANT
8425 TOE REATTACHMENT	856 MASTOPEXY
8426 FOOT REATTACHMENT	857 TOTAL BREAST RECONSTRUCT
8427 LOWER LEG/ANKLE REATTACH	8582 BREAST SPLIT-THICK GRAFT
8428 THIGH REATTACHMENT	8583 BREAST FULL-THICK GRAFT
8429 REATTACHMENT NEC	8584 BREAST PEDICLE GRAFT
843 AMPUTATION STUMP REVIS	8585 BREAST MUSCLE FLAP GRAFT
8440 IMPLNT/FIT PROS LIMB NOS	8586 TRANSPOSITION OF NIPPLE
8444 IMPLANT ARM PROSTHESIS	8587 NIPPLE REPAIR NEC
8448 IMPLANT LEG PROSTHESIS	8589 MAMMOPLASTY NEC
8458 IMP INTRSPINE DECOMP DEV OCT05-	8593 BREAST IMPLANT REVISION
8459 INSERT OTH SPIN DEVICE	8594 BREAST IMPLANT REMOVAL
8460 INSERT DISC PROS NOS (OCT 04)	8595 INSER BREAST TISSU EXPAN
8461 INS PART DISC PROS CERV (OCT 04)	8596 REMOV BREAST TISSU EXPAN
8462 INS TOT DISC PROST CERV (OCT 04)	8599 BREAST OPERATION NEC
8463 INS SPIN DISC PROS THOR (OCT 04)	8606 INSERT INFUSION PUMP
8464 INS PART DISC PROS LUMB (OCT 04)	8621 EXCISION OF PILONID CYST
8465 INS TOTL DISC PROS LUMB (OCT 04)	8622 EXC WOUND DEBRIDEMENT

8466 REVISE DISC PROST CERV (OCT 04)<br>864 RADICAL EXCIS SKIN LES<br>8660 FREE SKIN GRAFT NOS<br>8661 FULL-THICK HAND SKIN GRF<br>8662 HAND SKIN GRAFT NEC<br>8663 FULL-THICK SKIN GRFT NEC<br>8665 HETEROGRAFT TO SKIN<br>8666 HOMOGRAFT TO SKIN<br>8667 DERMAL REGENER GRAFT<br>8669 FREE SKIN GRAFT NEC<br>8670 PEDICLE GRAFT/FLAP NOS<br>8671 CUT & PREP PEDICLE GRAFT<br>8672 PEDICLE GRAFT ADVANCEMEN<br>8673 ATTACH PEDICLE TO HAND<br>8674 ATTACH PEDICLE GRAFT NEC<br>8675 REVISION OF PEDICLE GRFT<br>8681 REPAIR FACIAL WEAKNESS	8682 FACIAL RHYTIDECTOMY<br>8683 SIZE REDUCT PLASTIC OP<br>8684 RELAXATION OF SCAR<br>8685 SYNDACTYLY CORRECTION<br>8686 ONYCHOPLASTY<br>8689 SKIN REPAIR & PLASTY NEC<br>8691 SKIN EXCISION FOR GRAFT<br>8693 INSERT TISSUE EXPANDER<br>8694 INS/REPL SINGLE PUL GEN (OCT 04)<br>8695 INS/REPL DUAL PULSE GEN (OCT 04)<br>8696 INSERT/REPL OTH NEUROST 10/04<br>8697 INS/REP 1 PUL GEN OCT05-<br>8698 INS/REP 2 PUL GEN OCT05-<br>8753 INTRAOPER CHOLANGIOGRAM<br>9504 ANESTHETIZED EYE EXAM<br>8625 DERMABRASION
